# Generation of Functioning Nephrons by Implanting Human Pluripotent Stem Cell-Derived Kidney Progenitors

**DOI:** 10.1016/j.stemcr.2018.01.008

**Published:** 2018-02-08

**Authors:** Ioannis Bantounas, Parisa Ranjzad, Faris Tengku, Edina Silajdžić, Duncan Forster, Marie-Claude Asselin, Philip Lewis, Rachel Lennon, Antonius Plagge, Qi Wang, Adrian S. Woolf, Susan J. Kimber

**Affiliations:** 1 Division of Cell Matrix Biology and Regenerative Medicine, Faculty of Biology, Medicine and Health, University of Manchester, Manchester Academic Health Science Centre, Manchester, UK; 2 Royal Manchester Children's Hospital, Manchester, UK; 3 Division of Informatics, Imaging and Data Sciences, School of Health Sciences, Faculty of Biology, Medicine and Health, The University of Manchester, Manchester, UK; 4 Wellcome Trust Centre for Cell-Matrix Research, Division of Cell Matrix Biology and Regenerative Medicine, Faculty of Biology, Medicine and Health, University of Manchester, Manchester, UK; 5 Institute of Translational Medicine, University of Liverpool, Liverpool, UK

**Keywords:** human embryonic stem cells, kidney, nephron, glomerulus, lentivirus, kidney progenitors, metanephric mesenchyme, ureteric epithelium, vascularization, cell therapy

## Abstract

Human pluripotent stem cells (hPSCs) hold great promise for understanding kidney development and disease. We reproducibly differentiated three genetically distinct wild-type hPSC lines to kidney precursors that underwent rudimentary morphogenesis *in vitro*. They expressed nephron and collecting duct lineage marker genes, several of which are mutated in human kidney disease. Lentiviral-transduced hPSCs expressing reporter genes differentiated similarly to controls *in vitro*. Kidney progenitors were subcutaneously implanted into immunodeficient mice. By 12 weeks, they formed organ-like masses detectable by bioluminescence imaging. Implants included perfused glomeruli containing human capillaries, podocytes with regions of mature basement membrane, and mesangial cells. After intravenous injection of fluorescent low-molecular-weight dextran, signal was detected in tubules, demonstrating uptake from glomerular filtrate. Thus, we have developed methods to trace hPSC-derived kidney precursors that formed functioning nephrons *in vivo*. These advances beyond *in vitro* culture are critical steps toward using hPSCs to model and treat kidney diseases.

## Highlights

• Reproducible differentiation to kidney progenitors in 3 hESC lines
• After subcutaneous implantation, kidney-like tissues detectable by bioluminescence
• Implant nephrons contain glomeruli, proximal and distal tubules, and collecting ducts
• Vascularized glomeruli filter intravenously injected low-molecular-weight dextran

## Teaser

Kimber and colleagues show that pluripotent stem cell-derived kidney progenitors implanted subcutaneously generate vascularized glomeruli including podocytes with slit diaphragms and mature glomerular basement membranes indicative of functioning glomeruli. Human cells contributed to the vasculature, and the glomeruli were able to filter low-molecular-weight dextran injected intravenously, which appeared in some tubules.

## Introduction

The mammalian kidney generates and eliminates waste products and is essential for life. Annually, 2.6 million people worldwide receive dialysis or kidney transplantation for end-stage kidney disease (ESKD), while around 2.2 million people with ESKD die prematurely, unable to access treatment (Liyanage et al., 2015). Kidney transplants are in short supply and an adult on long-term dialysis has an average life expectancy of barely a decade (Neild, 2017). Therapies that prevent the progression of chronic kidney disease to ESKD are therefore urgently needed.

The definitive human kidney, the metanephros, initiates at 5 weeks of gestation (Woolf and Jenkins, 2015) when it is composed of metanephric mesenchyme (MM) around a ureteric bud (UB), both derived from intermediate mesoderm. Over the next month, MM differentiates to form the first nephrons, each containing a glomerulus in continuity with proximal and distal tubules. Concurrently, the UB branches serially to form collecting ducts that fuse with nascent nephrons. As the glomerulus matures, endothelia invade clusters of podocytes, forming capillary loops. The adjacent endothelia and epithelia are separated by the glomerular basement membrane (GBM), and the three components act as a functional unit that filters blood. The resulting ultrafiltrate is then modified by tubules to form definitive urine.

Given that kidney disease can result from genetic aberrations (Kerecuk et al., 2008, Hildebrandt, 2010) *in utero*, there is an urgent need to better understand the development of human kidneys. Although mouse models have been informative about developmental mechanisms (Woolf and Davies, 2013, McMahon, 2016), they do not always exactly phenocopy human kidney diseases that result from mutations of homologous genes (Woolf and Jenkins, 2015, Kerecuk et al., 2008). Clearly, human models are the ideal systems for understanding organogenesis in relation to human health and disease.

The use of expandable human pluripotent stem cells (hPSCs) with their plasticity in response to developmental signals is a promising and logical choice for many disease therapies (Carpenter et al., 2009, Cheng et al., 2014, Ichimura and Shiba, 2017) and for modeling monogenic diseases (Ebert et al., 2009, Shi et al., 2017). This includes making kidney cells to model ESKDs, or for therapy. Several *in vitro* studies have demonstrated that a defined cocktail of growth factors and small molecules, applied in a timed sequence to hPSCs, can result in primitive kidney morphogenesis in 2D (Narayanan et al., 2013, Kang and Han, 2014, Lam et al., 2014, Takasato et al., 2014) and in 3D (Xia et al., 2014, Takasato et al., 2015, Ciampi et al., 2016) cultures. This led to the development of immature kidney structures, allowing interaction between UB and MM tissues and their co-operative development. This technology is beginning to show promise to model both genetic (Freedman et al., 2015) and acquired (Morizane et al., 2015) kidney diseases. Questions remain, however, regarding the reproducibility of the differentiation protocols, replicability between hPSC lines, and the degree of maturity and function that can be obtained. In 3D transwell formats, kidney structures progress further giving some regional organization (Takasato et al., 2015), but the kidney progenitors are necessarily limited in their growth and functional differentiation because, for example, they lack a blood supply.

With these limitations in mind, we used three wild-type hPSC lines from different genetic backgrounds and reproducibly differentiated them into kidney progenitors *in vitro*. They underwent rudimentary morphogenesis and expressed MM/nephron and UB/collecting duct lineage markers. Using bicistronic lentiviral reporters to trace the hPSC derivatives *in vivo*, we showed that the kidney progenitors formed functional nephrons following subcutaneous implantation. Compared with *in vitro* culture, hPSC-kidney differentiation was dramatically improved with the generation of glomeruli, containing human capillaries and podocytes separated by regions of mature basement membrane. These are critical advances toward using hPSCs to model and treat kidney diseases.

## Results

### Gene Expression Patterns in hPSCs Induced to Form Kidney Precursors in Culture

**Figure 1:**
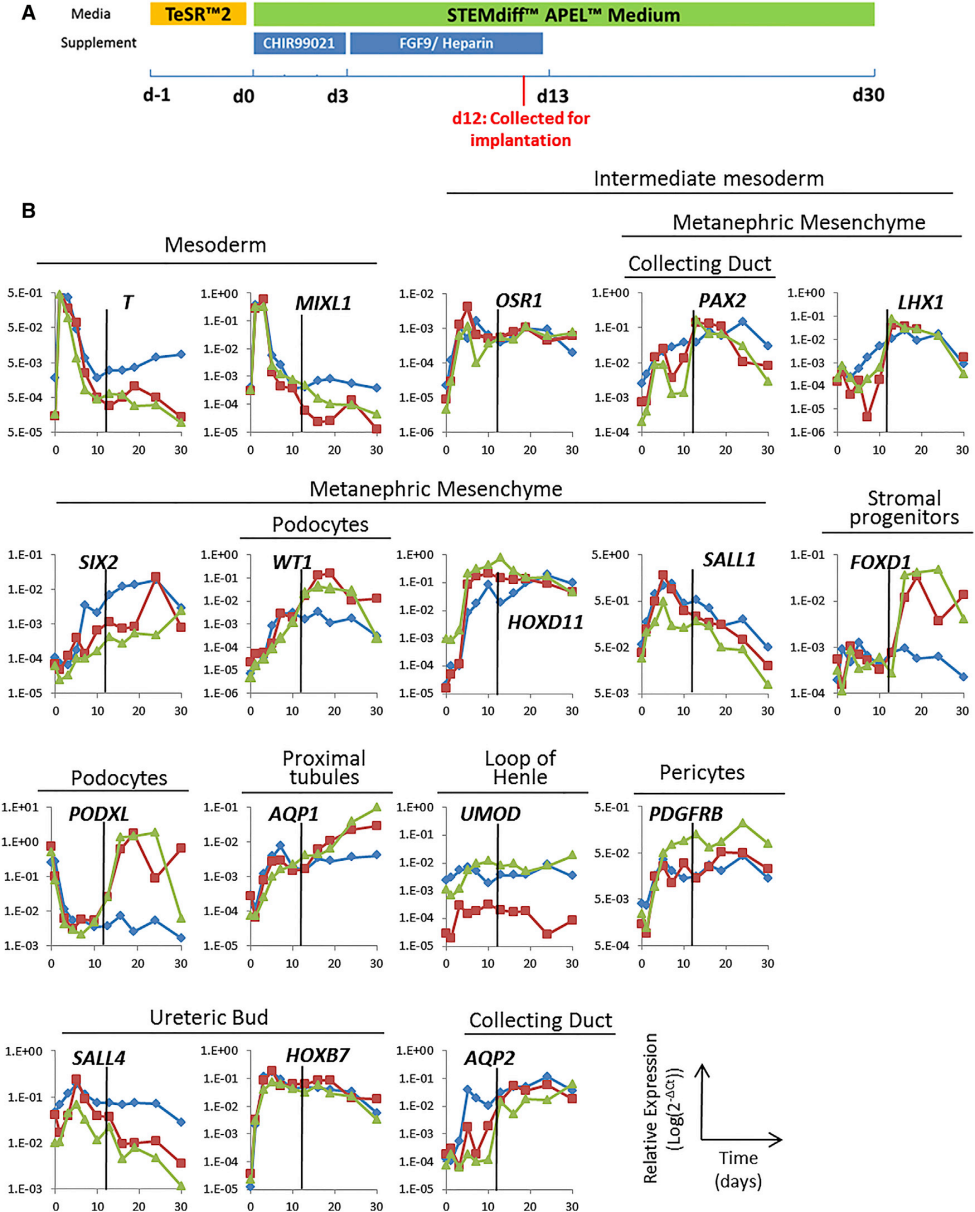
Differentiation of MAN13 hPSC to Kidney Lineages in 2D Culture (A) Schematic of the 30 day differentiation protocol depicting the timing of application of CHIR99021 and FGF9/heparin. The time point of cell harvest for implantation into mice is also indicated (*d12*, in red). (B) qPCR profiling of 17 transcripts at 11 time points over 30 days. The results for three independent experiments are shown in blue, red, and green, with levels of target transcripts normalized to *GAPDH* expression. The characteristic tissue/lineage that expresses each gene *in vivo* is indicated above the graph for each transcript. The black vertical line in each graph indicates the time of collection of cells for implantation into mice.

To obtain kidney progenitor cells for transplantation, we first determined whether three characterized human embryonic stem cell (hESC) lines, clinical grade MAN13, MAN11 (Ye et al., 2017, Canham et al., 2015), and HUES1 (Cowan et al., 2004, Oldershaw et al., 2010) had the potential to differentiate into kidney progenitors using an established protocol (Takasato et al., 2014). This comprised exposure to CHIR99021, a glycogen synthase kinase-3 inhibitor, for 3 days before switching to FGF9 and heparin for 10 days, followed by basal STEMdiff APEL medium alone until day 30 (Figure 1A). Using qPCR, we documented the expression of 17 key transcripts characterizing mesoderm, intermediate mesoderm, MM and its nephron segment derivatives, and the UB and its collecting duct derivatives.

In three separate experiments with MAN13, transcripts for *T* (*Brachyury*) and *MIXL1*, mesodermal/mesendodermal transcription factors, peaked 1 day into the protocol (Figure 1B). As these were downregulated, *OSR1* and *PAX2*, intermediate mesoderm transcription factor markers, were upregulated: *PAX2* is also expressed in UB/collecting ducts and MM, and *OSR1* in MM. The expression of both transcripts was maintained during the rest of the *in vitro* protocol with a slight decrease in *PAX2* toward day 30. In the first 7–10 days, transcripts for a battery MM/nephron lineage transcription factors (*LHX1*, *SIX2*, *WT1*, *HOXD11*, and *SALL1*) increased, with an early peak in *SALL1* and *HOXD11* and subsequent robust *SIX2* and *WT1* expression. Up to day 30, there was a progressive increase in levels of *AQP1*, encoding a proximal tubule water channel, with variable upregulation of *PODXL*, encoding a podocyte sialomucin, between days 10 and 20. Transcripts for *PDGFRB*, a marker of pericytes and required for endothelial development, increased up to day 20, while those for *FOXD1*, a kidney stromal progenitor and endothelial development marker, increased after day 10. *SALL4* and *HOXB7,* transcription factors of the UB lineage, were induced in the first week of the protocol, whereas *AQP2*, which encodes a collecting duct water channel, rose progressively up to day 30. *UMOD* transcripts, marking the thick ascending limb of the loop of Henle, were also detected during differentiation. Similar patterns of transcript expression were recorded in HUES1 and MAN11, exposed to this differentiation protocol (Figure S1). These results suggested reproducibility of the protocol for obtaining kidney cells from different hESC lines, and we focused on one, MAN13, for the rest of the study.

### Rudimentary Morphogenesis by hPSC-Derived Kidney Precursors in 2D Culture

**Figure 2:**
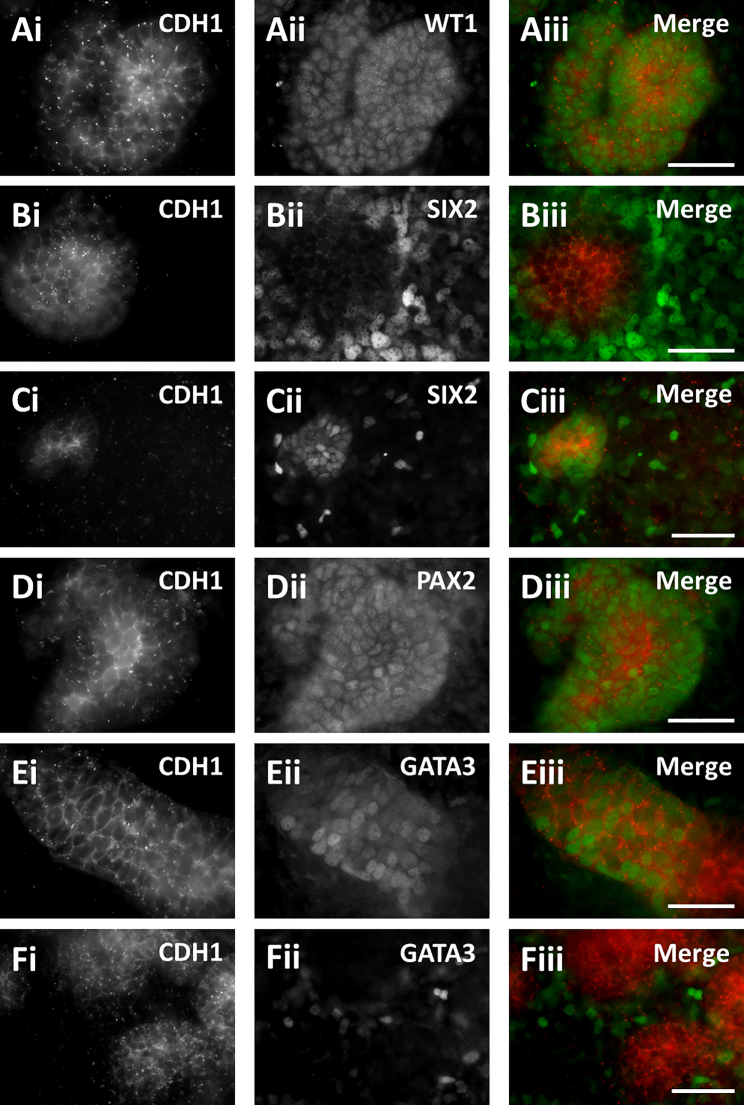
Immunocytochemistry of hPSC Cultures MAN13 cells were differentiated for 12 days *in vitro*, when rudimentary morphogenesis had begun. Cultures were co-stained with the epithelial cell-cell adhesion protein CDH1 (left frames) and transcription factors (middle frames) to detect: MM-derived cells (WT1 or SIX2); UB-derived tubules (GATA3); and PAX2, expressed in both lineages. Right-hand frames show merged images (red CDH1 and green nuclear proteins). (A) A WT1+ cell cluster with a central zone expressing CDH1. (B) Typically, loose SIX2+ cells surrounded CDH1+ zones. (C) Occasionally, subsets of SIX2+ cells appeared to co-express CDH1. (D) A PAX2+ cluster with a central zone expressing CDH1. (E) A CDH1+ tubule-like structure containing a subset of GATA3+ nuclei. (F) In other areas, loosely packed GATA3+ cells surrounded CDH1+ structures. Scale bars, 40 μm.

On day 12 of the 2D protocol, cultures comprised confluent lawns, interspersed with zones of increased cell density (Figure 2). We immunostained cultures for transcription factors expressed by MM/nephron (WT1, SIX2, and PAX2) and UB/collecting duct (GATA3 and PAX2) lineages, and for the epithelial adhesion protein CDH1 (E-cadherin). We observed WT1+ cell clusters, some with CDH1+ cores (Figure 2A). *In vivo*, glomerular podocytes, as well as induced MM, express WT1 but immunostaining cultures for the podocyte marker nephrin, and for the distal convoluted tubule marker TRPV5, proved negative (data not shown). SIX2+ cells were mostly detected in loose populations surrounding CDH1+ structures (Figure 2B), although we occasionally observed CDH1+/SIX2+ clusters (Figure 2C), likely representing initial epithelialization within the MM/nephron lineage. PAX2+ clusters were visualized, some containing CDH1+ cores (Figure 2D). For GATA3, two patterns were observed: first, some CDH1+ tubule-like structures contained subsets of GATA3+ cells (Figure 2E), consistent with a collecting duct identity; the other pattern comprised scattered GATA3+ cells around CDH1+ aggregates (Figure 2F), as described in a similar protocol (Takasato et al., 2015). After 30 days of differentiation *in vitro*, more complex cell clusters were seen (Figure S2). Even at this more advanced stage, SIX2+ cells were present, consistent with potential for further nephrogenesis (Kobayashi et al., 2008). However, because MM and UB progenitor populations were clearly present at day 12, we reasoned that this would be a suitable point to test their further differentiation after implantation *in vivo*.

### hPSC Differentiation in 3D Organoid Cultures

Aiming to further enhance glomerular morphogenesis we evaluated alternative protocols. Takasato et al. (2015) differentiated hESCs for up to a week in 2D culture as above, but then subjected them to a pulse of CHIR99021, after pelleting and placing at a medium-air interface. We replicated their formation of 3D organoids with more mature kidney structures compared with 2D culture (Figure S3), but, although glomerular structures were formed, they were immature and not identical to mature glomeruli *in vivo.* In particular, glomerular tufts lacked capillaries and did not express mature collagen IV. We reasoned that maturation may require more time and factors in the *in vivo* environment and set out to evaluate kidney development *in vivo*.

### Lentivirus-Mediated Transduction of hPSCs with Reporter Genes

**Figure 3:**
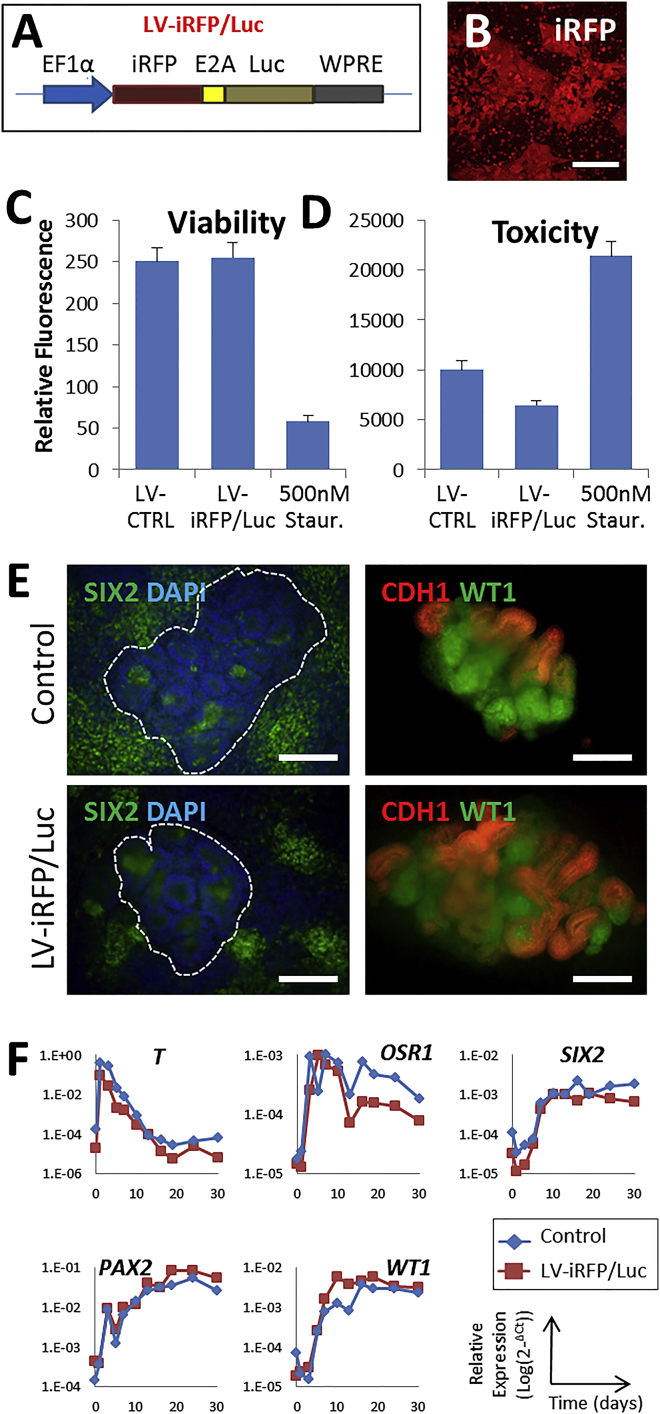
Transduction of MAN13 hPSCs with a Lentiviral Vector Expressing a Bicistronic *iRFP-E2A-*Luciferase Cassette (A) Diagram of the expression cassette showing the two reporter genes and the *EF1α* promoter. (B) iRFP fluorescence in transduced cells. (C and D) Viability and cytotoxicity (mean ± SEM, n = 4) in MAN13 cultures transduced with lentivirus (*LV-iRFP/Luc*), with no significant difference compared with untransduced controls (*LV-CTRL*). As a positive “death” control, MAN13 cells were treated with 500 nM staurosporine for 24 hr. (E) Examples of transduced and untransduced differentiating MAN13 cultures assessed by immunocytochemistry for SIX2, WT1, and CDH1. (F) Similar qPCR profiles of transduced versus parent cells during 2D kidney differentiation. A representative experiment of three independent biological repeats is shown for each line. Scale bars, 200 μm in (B) and 90 μm in (E).

To trace implanted kidney progenitors *in vivo*, we generated an integrating lentiviral vector carrying a bicistronic cassette, expressing a near infrared fluorescent protein (iRFP, emission at 720 nm) and firefly luciferase, both under the control of the *EF1α* promoter (Figure 3A). Transduction of MAN13 hESCs resulted in robust expression of the fluorescent protein (Figure 3B). Transduction did not affect viability nor was it toxic (Figures 3C and 3D). Furthermore, up to day 30 of the *in vitro* protocol, both the parent and transduced lines showed similar morphogenesis (Figure 3E) and patterns of transcript for *T*, *OSR1*, *SIX2*, *PAX2*, and *WT1* (Figure 3F).

### Assessment of Pluripotency in Lentivirus-Transduced hESCs by Teratoma Assay

To determine whether lentiviral labeling compromised the pluripotent potential of hPSCs we subcutaneously implanted MAN13 parent hESCs and transduced MAN13 hESCs in the backs of *SCID/beige* mice as aliquots of one million cells in Matrigel. Mice were culled when an implanted cell mass reached a maximum 1.6 cm across (Table S1). This protocol led to mice being culled at similar times (parent line, 38.8 ± 1.2 days; transduced line, 44.6 ± 1.2 days; mean ± SEM; p = 0.07, Student's t test). Each line was similarly efficient at forming histologically confirmed masses (20 out of 24 sites in the parent versus 29 out of 36 in the transduced cells; p = 1.00, Fisher's exact test, two-tailed) that were of similar sizes (parent line, 1.13 ± 0.07 cm versus transduced line, 0.98 ± 0.07 cm; p = 0.11, Student's t test). Histology revealed that all masses had hallmarks of teratomas (Figure S4A), containing endodermal (Figure S4B), mesodermal (Figure S4C), and ectodermal (Figure S4D) derivatives. Teratomas formed from lentivirus-labeled cells were detectable using bioluminescent imaging of living mice (Figure S4E), and expressed luciferase (Figure S4F) and human mitochondrial antigen (Figure S4G), confirming their hESC origin.

### Subcutaneous Implantation of Kidney Progenitor Cells Derived from Labeled hPSCs

Since lentiviral labeling of MAN13 hPSCs had no detrimental effect on hESC pluripotency, self-renewal, or differentiation to kidney progenitors in 2D culture, we reasoned that labeled kidney precursor cells generated in 2D culture would be suitable for further differentiation *in vivo*. Based on our *in vitro* data, we reasoned that progenitors on day 12 of 2D differentiation would be committed to kidney lineages but still able to respond in the *in vivo* environment, while having lost the potential to form teratomas. Therefore, on day 12, lentivirus-labeled MAN13-derived kidney progenitors were injected subcutaneously into *SCID/beige* mice, as for hESCs. Mice were followed for up to 12 weeks, at which time no mouse had an implant site with a cellular mass of 1.6 cm. In fact, masses were only rarely detected on external inspection and palpation, leading to harvesting at 84.3 ± 0.2 days (Table S1), significantly longer (p < 0.001, Student's t test) than for the hESC implants. Only 9 of 32 implant sites contained histologically identified masses, significantly fewer than for undifferentiated hESC implants (p < 0.001, Fisher's exact test, two-tailed). Moreover, these masses were an average diameter of 0.61 ± 0.11 cm, significantly smaller (p < 0.05, Student's t test) than for hESC-derived teratomas.

**Figure 4:**
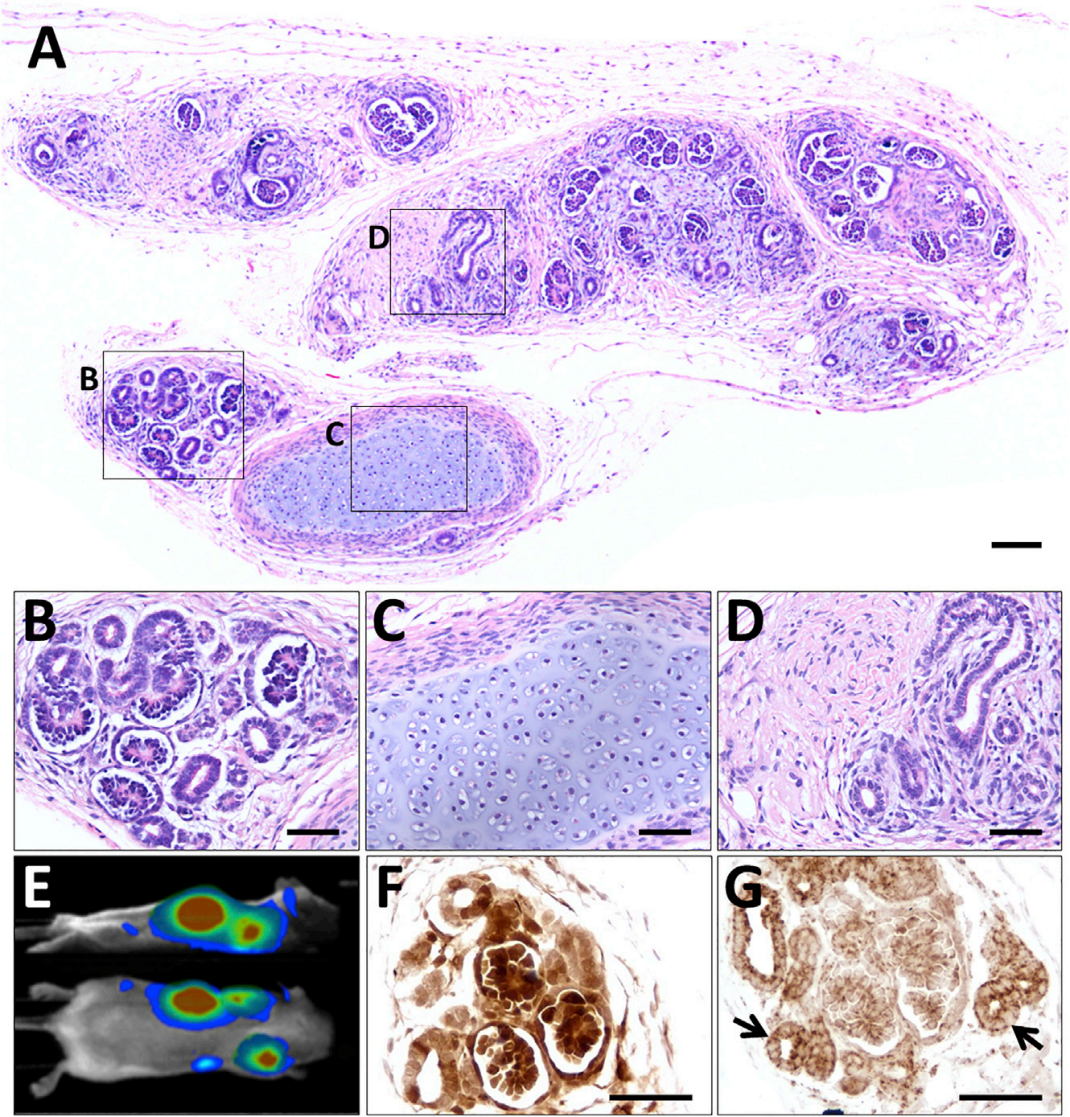
Subcutaneous Implantation into *beige/SCID* Mice of Luciferase-Labeled MAN13-Derived Kidney Precursor Cells Harvested at Day 12 in 2D Culture (A–D) Histological overview 12 weeks after transplantation. The implanted cells have formed a differentiated mass (A). Boxed areas indicate the following: (B) differentiated nephrons; (C) cartilage; and (D) poorly differentiated tubules and stroma. (E) Side and dorsal views showing bioluminescence in a living mouse that had received kidney precursor transplants 12 weeks previously. (F) Immunostaining (brown) for luciferase in area containing nephron-like structures. (G) Immunostaining (brown) for human mitochondria, arrows indicate tubules. Sections in (F) and (G) are not counterstained. Scale bars, 100 μm (A) and 50 μm in (B)–(D), (F), and (G).

Histology of tissues generated from transplanted MAN13 kidney precursors (Figure 4A) was strikingly different from that of teratomas. The kidney precursor implants comprised differentiated kidney structures with glomeruli and tubules (Figure 4B), as well as occasional islands of cartilage (Figure 4C) and poorly differentiated metanephric tissues (Figure 4D). The masses detected at autopsy corresponded to the bioluminescent areas detected in living mice using non-invasive imaging (Figure 4E), and reacted with antibodies to luciferase (Figure 4F) and human mitochondrial antigen (Figure 4G) in sections, confirming that they were composed of human cells. There was some variation in the degree of differentiation between implants: for example, the mass in Figure S5 contained prominent zones in which isolated glomeruli were surrounded by mesenchyme-like cells and primitive tubules. From comprehensive histological analysis, of all nine masses derived from implanted kidney progenitors, we concluded that teratomas were not generated and that the cells were able to survive and continue to differentiate to kidney tissue.

Next, we implanted a second group of mice with MAN13 cells, some differentiated for 12 days, and others for 19 days, using the *in vitro* 2D protocol. The day 12 implants formed glomeruli and tubules, similar to those in the first study, above, at 7/20 injected sites. Although not visible externally, the day 19 implants resulted in a few small masses observed by luminescence in a similar proportion 8/20, injected sites at 12 weeks after implantation (Table S1). These exhibited an immature morphology (Figure S6) compared with those generated from day 12 progenitors. This suggests there is likely to be a critical stage of kidney differentiation in culture beyond which dissociated cells lose their plasticity and ability to respond to an *in vivo* environment, or to each other and undergo only limited nephrogenesis.

### Detailed Phenotyping of Implant Tissues from hPSC-Derived Kidney Precursor Cells

**Figure 5:**
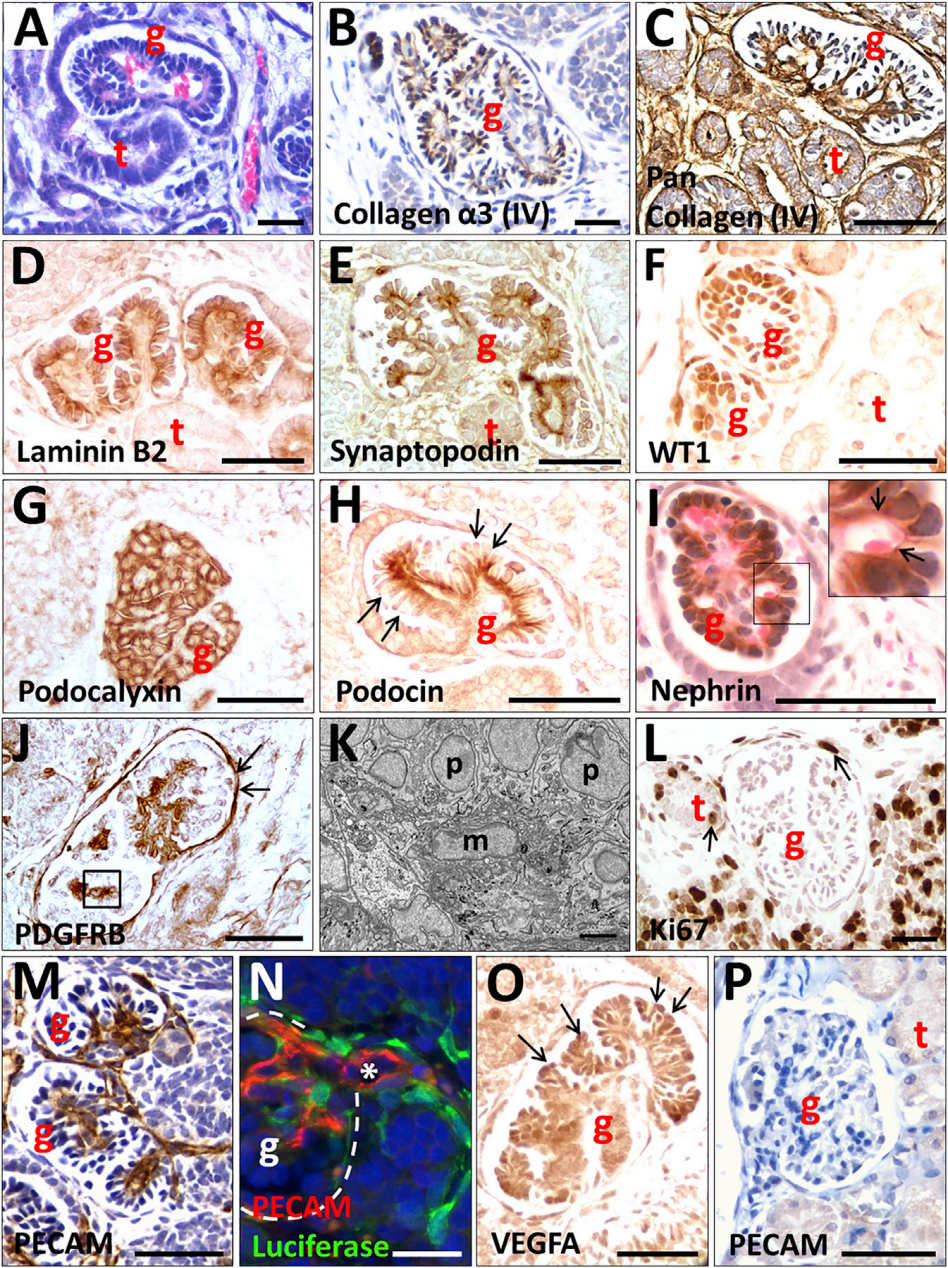
Histology of Glomeruli Generated from Implanted Luciferase-Labeled MAN13-Derived Kidney Precursor Cells Harvested at Day 12 of 2D Culture Images in (A)–(O) are implants, whereas (P) is an adult mouse glomerulus. (A) and (I) were counterstained with H&E; (B), (C), (M), and (P) were counterstained with hematoxylin only; other sections were not counterstained. All frames are bright-field views apart from (K), which is TEM and (N), which is epifluorescence. g indicates a glomerulus, t indicates a tubule, p indicates a podocyte, and m indicates a mesangial cell. (A) Glomerulus with red blood cells in its tuft. Note the lumen of the tubule in continuity with the Bowman space of the glomerulus. (B) Collagen α-3 (IV) immunostaining (brown) in a GBM-like pattern. (C) Pan-collagen IV immunostaining (brown) in a glomerulus and nearby tubules. (D) Laminin B2 (brown) immunostaining in two glomeruli but negligible in the tubule. (E) Synaptopodin IHC (brown) in a linear pattern on the basal aspect of the podocytes. (F) WT1 IHC (brown) in podocyte nuclei. (G) Podocalyxin IHC (brown) in podocytes. (H) Podocin immunostaining (brown) in a linear pattern at the basal side of podocytes (arrows indicate apical sides of podocytes). (I) Nephrin immunostaining (brown) in a glomerulus. The boxed area is enlarged on the top right corner of the frame: arrows indicate nephrin in a linear pattern adjacent to a capillary loop containing a red blood cell. (J) Platelet-derived growth factor receptor B (PDGFRB) IHC (brown) in the center of a glomerular tuft where mesangial cells reside (boxed area). Arrows indicate additional immunostaining in Bowman capsule. (K) TEM of a similar area as depicted by a box in (J). m marks a mesangial-like cell and p indicates podocytes. (L) Ki67 immunostaining (brown) marking proliferation in more poorly differentiated cells near a glomerulus and a tubule; occasional positive nuclei (arrows) were detected in tubules and Bowman capsules. (M) PECAM immunostaining (brown) shows an extensive capillary network in glomerular tufts. (N) PECAM (red) and luciferase (green) double immunostaining. The white asterisk marks the lumen of a small artery that is continuous with the capillary network in the glomerular tuft; the white dotted line marks the Bowman capsule. Note that luciferase-expressing cells are closely associated with endothelia whose luminal surface is positive for PECAM. (O) VEGFA immunostaining (brown) was prominent in podocytes (arrows). (P) Mouse glomerulus is not reactive with the anti-human PECAM monoclonal antibody. Scale bars, 50 μm in (A)–(J) and (L)–(P) and 500 nm in (K).

Next, we characterized the kidney structures formed within the implants. Glomeruli contained red blood cells in their tufts, consistent with a blood supply, and Bowman spaces were observed continuous with tubules (Figure 5A). Glomeruli were immunoreactive with antibodies raised against the mature GBM proteins, collagen α-3 (IV) and laminin β2, and to anti-pan-collagen IV, which was also reactive with basement membranes of nearby tubules (Figures 5B–5D). Podocyte-like cells in glomerular tufts were detected with antibodies to synaptopodin, WT1, and podocalyxin (Figures 5E–5G). Podocin (Figure 5H) and nephrin (Figure 5I), podocyte slit diaphragm proteins, were prominent on the basal aspect of podocytes. Platelet-derived growth factor receptor B staining was present in the center of the glomerular tuft where mesangial cells reside (Figure 5J). Mesangial-like cells were apparent in this location by transmission electron microscopy (TEM) (Figure 5K and Movie S1). Ki67, was rarely detected in glomeruli, but proliferative cells were prominent in nearby less differentiated areas (Figure 5L).

An antibody against a human PECAM epitope revealed networks of capillaries within tufts of the majority of glomeruli (Figure 5M). We also detected larger, arteriole-like, vessels near glomeruli within the implant. Using double immunostaining, luciferase-expressing rhomboidal pericyte-like cells were closely associated with PECAM+ endothelia in these vessels (Figure 5N). In addition, glomerular podocytes were positive for the vascular growth factor VEGFA (Figure 5O). Notably, the PECAM antibody did not react with mouse kidney sections (Figure 5P). Thus implant glomeruli are vascularized, and endothelia in the glomerular tufts, and vessels around the glomeruli, can derive from implanted human cells. Moreover, the day 12 kidney precursor-derived tissues *in vivo* are morphologically and molecularly substantially more mature than those formed in 3D organoid cultures (compare Figure 5 with Figure S3). *In vivo*, the glomeruli expressed all the markers tested and, crucially, they were vascularized in a pattern similar to native glomeruli *in vivo*, unlike in the *in vitro* 3D organoid cultures.

**Figure 6:**
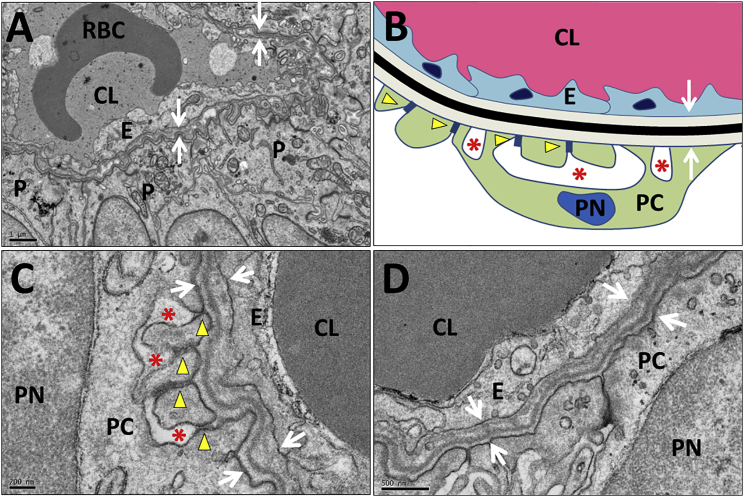
TEM of a Glomerulus Generated from Implanted MAN13-Derived Kidney Precursor Cells (A) TEM overview of a capillary lumen (CL) containing a red blood cell (RBC) and lined by endothelial cells (E). Podocyte-like cells (PC) and endothelia abut a shared basement membrane (white arrows). (B) Diagram of ultrastructure of a mature glomerulus showing the spatial relationships between the capillary lumen (CL, red), endothelial cells (E, light blue), and podocytes (PN, dark blue, is a podocyte nucleus, and PC, green, is podocyte cytoplasm). Endothelia and podocytes rest on a shared trilaminar GBM, with the central lamina densa (black) flanked by the lamina rara interna on the endothelial side and lamina rara externa on the podocyte side (both light grey). Yellow arrows indicate electron dense slit diaphragm like structures joining podocyte foot processes that abut the GBM. Asterisks indicate spaces between the foot processes that receive glomerular ultrafiltrate. (C) High-power TEM showing mature organization of trilaminar GBM (between white arrowheads) and dark slit diaphragm-like structures (yellow arrowheads) between podocyte foot processes. Asterisks indicate urinary spaces between foot processes. (D) TEM of another zone showing a less mature appearance. This GBM has two dark lamina densae (white arrow), as occurs in nascent glomeruli. Scale bars, 1 μm in (A), 200 nm in (C), and 500 nm in (D).

**Figure 7:**
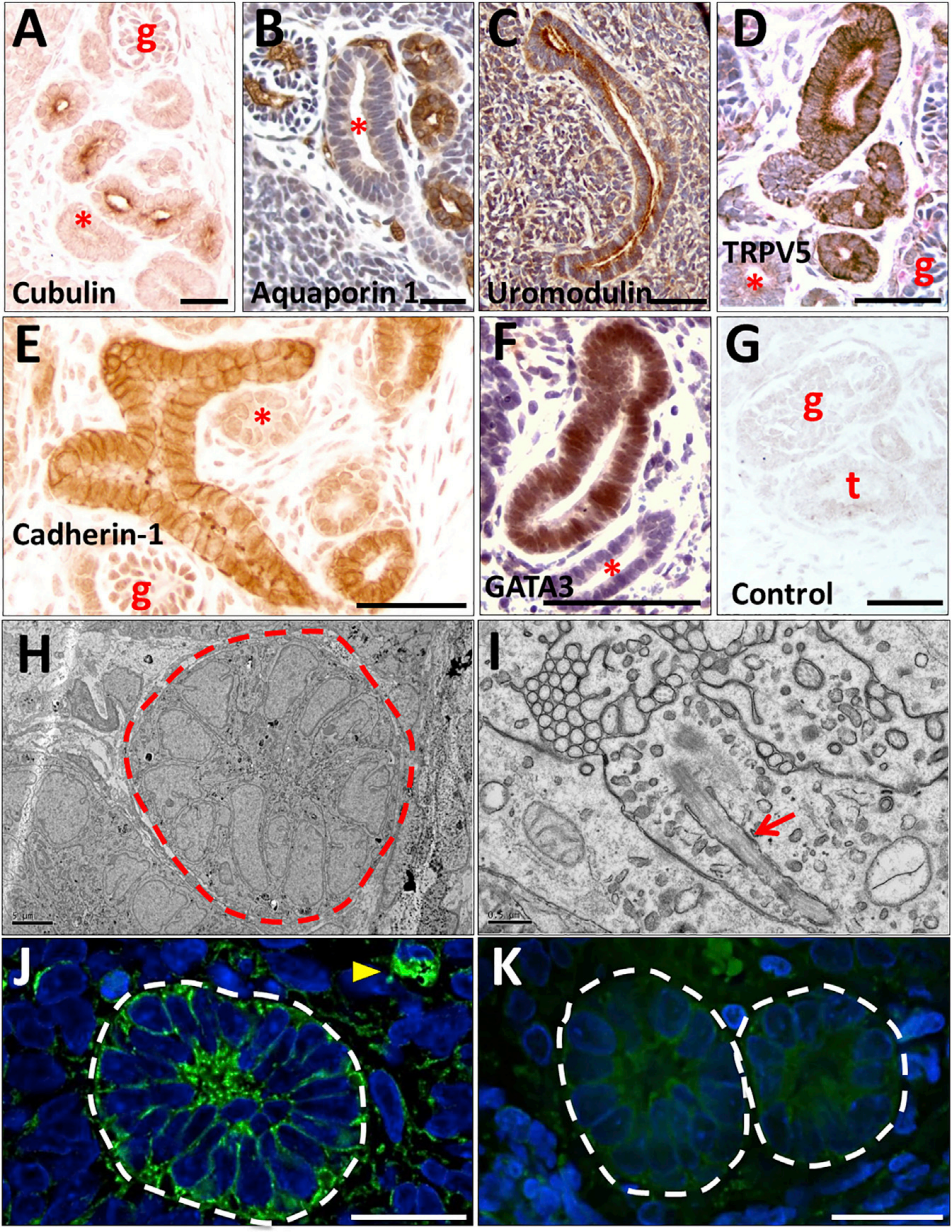
Tubules Formed from Implanted MAN13-Derived Kidney Precursor Cells and Evidence of Nephron Functionality (A)–(G) show bright-field IHC, with (B), (C), (D), and (F) counterstained with hematoxylin. (H) and (I) are TEM images. (J) and (K) are epifluorescence imaging. (A) Brush border-like immunostaining (brown) with antibody to cubulin, a proximal tubule protein, in a subset of tubules. A negative tubule is marked with a red asterisk. (B) Aquaporin 1 immunostaining (brown) in a subset of tubules; another (asterisk) is negative. Note, as expected, aquaporin 1 is also present in glomerular and interstitial capillaries. (C) Immunostaining for uromodulin (brown), a protein in thick ascending limbs of loops of Henle. (D) Immunostaining for TRPV5 (brown), a protein that marks distal convoluted tubules; another tubule (asterisk) and glomeruli are negative. (E) CDH1 immunostaining (brown) in large branched tubules. (F) GATA3 immunostaining (brown) in a large tubule; the smaller tubule (asterisk) is negative. (G) Negative control: rabbit secondary antibody applied but primary antibody omitted. (H) Overview TEM image of a cross-section of a tubule, outlined in red dashes. (I) High-power TEM of central zone of tubules showing a primary cilium (red arrow); above its basal body is a zone with cross-sections of a cluster of microvillus-like structures. (J) Section from a kidney progenitor-derived mass harvested from a mouse intravenously injected with low-molecular-weight, fluorescein isothiocyanate (FITC)-labeled dextran. White dashes surround a cross-section of a tubule containing green fluorescence, most marked in its apical, central, zone. The yellow arrow indicates a small blood vessel that itself contains injected FITC-dextran. (K) An equivalent section from an implant in a mouse not injected with FITC-dextran shows background green fluorescence only. Scale bars, 50 μm in (A)–(G) and (J)–(K), 5 μm in (H), and 0.5 μm in (I).

TEM revealed that glomeruli in implants possessed a characteristic arrangement of podocytes on the outer surface of blood capillaries (Figure 6A). Those capillaries often contained red blood cells, providing further evidence that the glomeruli are connected to the blood supply of the host. Higher-power TEM images of podocyte-capillary interfaces revealed that some had an ultrastructure indistinguishable from that seen in mature glomeruli (Figure 6B), with podocytes possessing the characteristic foot processes joined by slit diaphragm-like structures and urinary spaces visible under the foot processes (Figure 6C). Also present was a fused GBM (Figure 6C). Other areas, showed a less-mature GBM, sometimes double layered with two lamina rarae (Figure 6D), as occurs in glomerulogenesis (Sariola, 1984). Implant tissues also contained tubules, some being positive for nephron-segment markers: cubulin (Figure 7A) and aquaporin 1 (Figure 7B) in proximal-like tubules, uromodulin in thick ascending loops of Henle (Figure 7C), and TRPV5 in distal convoluted-like tubules (Figure 7D). Larger-diameter branching tubules expressed CDH1 (Figure 7E), and others GATA3, suggesting that they were collecting ducts (Figure 7F). TEM revealed microvilli and primary cilia in the tubules (Figures 7H and 7I).

Since human glomeruli with patent, perfused capillary loops were present, we investigated whether glomerular filtration might be occurring, using day 12 kidney precursor cells with tissue harvested after 11 weeks. Some mice were intravenously administered fluorescein isothiocyanate-labeled 10 kDa dextran 1 hr prior to culling, as this is filtered by glomeruli and then reclaimed by proximal tubules (Woolf et al., 1990). Histology revealed a subset of tubules with fluorescence, most marked in the apical zone (Figure 7J), with negligible background fluorescence in uninjected mouse implants (Figure 7K).

## Discussion

In this study, we have demonstrated that different hPSC lines can be reproducibly induced to form kidney precursors *in vitro*, which can further develop *in vivo* to generate mature kidney structures, with vascularized glomeruli that can filter blood to make ultrafiltrate that is processed by adjacent tubules.

Variation in outcome between lines and protocols has been an issue with protocols for obtaining PSC kidney cells in culture. We showed that three wild-type hESC lines exhibited consistent differentiation in 2D culture, despite different genetic backgrounds (Ye et al., 2017). Furthermore, we labeled hPSCs with reporter genes for lineage tracing without compromising their differentiation potential, allowing hESC-derived kidney progenitor cells to be traced using non-invasive bioluminescence imaging after implantation into mice where they form functioning nephrons.

As shown here and previously (Takasato et al., 2015), 3D transmembrane organotypic cultures can modestly advance kidney morphogenesis from PSCs beyond 2D cultures. However, by implanting differentiating cells from 2D cultures *in vivo* we have substantially improved their maturity. Implanted progenitors survived subcutaneously for 3 months, forming markedly more mature kidney structures than reported previously. Notably, mature vascularized glomeruli were observed. Evidence for the maturity of these glomeruli comes from the observations that they expressed mature GBM proteins, laminin β2 and collagen α-3 (IV) (St John et al., 2001), with a fused trilaminar structure, as well as podocyte processes and slit diaphragms. Type IV collagen is an essential BM component and forms three distinct networks from combinations of six different α chains. Networks formed from trimers of α1,1,2 predominate in early mouse glomerular development, and there is an isoform switch to the α 3,4,5 network, which is most abundant in mature GBM (Harvey et al., 1998). Using α chain-specific antibodies we detected collagen α-3 (IV) in implant glomeruli indicative of assembly of the mature α 3,4,5 network. The detection and distribution of both podocin and nephrin at the basal aspect of podocytes within differentiated implants supports the conclusion that slit diaphragms were maturing within these glomeruli. Furthermore, the functionality of PSC-derived whole nephrons has been demonstrated *in vivo*: we detected uptake by tubule cells of low MW, filterable, fluorescent dextran injected into the host circulation. This level of maturity is remarkable given that the progenitor cells were implanted at an ectopic site, showing that PSC kidney development, such as embryonic/fetal kidney development, exhibits a high level of autonomy. This may be an advantage for future use of PSC kidney progenitors therapeutically, since they require limited instructive signaling from their environment in order to develop and may be more likely to develop and function normally in a suboptimal environment.

A key event in glomerulogenesis, prerequisite for delivery of blood for filtration, is invasion of the glomerular tuft by endothelia that form capillary loops. We observed organoids rich in glomeruli with WT1+ tufts in hPSC-derived kidney precursors maintained in 3D organ culture. Although interstitial spaces around the glomeruli contained PECAM+ cells, endothelia were rarely detected in glomerular tufts, consistent with the observations of Takasato et al. (2015). In contrast, we found that glomeruli formed after subcutaneous implantation of hESC-derived kidney precursors, had prominent capillaries, some containing red blood cells, consistent with perfusion. The human origin for PECAM+ glomerular capillaries in implants was confirmed by reactivity with the human-specific PECAM antibody, not seen in mouse kidney. We cannot exclude the possibility that a proportion of endothelia within implants originate from the host, and indeed connections between the two vasculatures must exist for perfusion to take place. We found that the glomeruli that formed in 3D organ culture had only diffuse low expression of VEGFA, whereas prominent immunostaining was detected in podocytes of glomeruli formed by implanted cells, supporting the conclusion from mice, that VEGFA is needed for glomerular maturation (Tufró, 2000, Eremina et al., 2003). Notably, glomeruli formed from implants also contained mesangial-like cells which provide mechanical integrity to capillary loops *in vivo*.

The key function of the kidney is to filter blood, generating an ultrafiltrate that is modified by tubules to form definitive urine. Our implants established the essential components for filtration: blood-perfused capillaries, podocytes, and regions of mature GBM between these cells. After intravenous injection of fluorescently labeled low-MW dextran glomerular filtration followed by tubule uptake of fluorescent dextran in a subset of PSC kidney tubules was observed. Others have transplanted intact human embryonic metanephric kidneys, but not PSC-derived kidney tissue, into immunodeficient mice where they differentiated into urine producing kidneys (Dekel et al., 2002, Dekel et al., 2003). Other studies have also attempted to generate functional glomeruli from human SCs. Xinaris et al. (2016) mixed human amniotic SCs with mouse metanephric kidney cells to form chimeric organoids and transplanted these into mice: human cells contributed to the formation of some functional glomeruli, assessed by the uptake of infused BSA. Using hPSCs carrying a podocyte-specific promoter-reporter gene, Sharmin et al. (2016) generated podocytes and mixed them with a human endothelial cell line. After transplanting the mixture under the kidney capsule, they reported the presence of vascularized glomeruli containing donor-derived podocytes. In neither study did a whole kidney-like organ form from a single source of human cells, as we have demonstrated for hPSCs.

The technological advances we have generated will facilitate use of hPSC-derived kidney tissues as models of genetic human disease. As the hPSCs differentiate toward kidney progenitors *in vitro*, they expressed the transcription factors PAX2 and SALL1, respectively mutated in the human renal coloboma (Sanyanusin et al., 1995) and Townes-Brocks (Faguer et al., 2009) syndromes featuring kidney malformations. We show that glomerular proteins that can be mutated in blood filtration diseases were present in implant glomeruli. These genetic diseases include congenital nephrotic syndrome (nephrin and podicin; Hildebrandt, 2010), Alport syndrome (collagen genes, including *COL4A3*; Lemmink et al., 1994), Pierson syndrome (*LAMB2*; Zenker et al., 2004), and Frasier syndrome (*WT1*; Barbaux et al., 1995). Other proteins essential for terminal differentiation of kidney tubules were also expressed in the implants: cubulin present in the apical brush border of proximal tubules, where it functions with megalin in uptake of small proteins; uromodulin, an apical protein in ascending loops of Henle and mutated in medullary cystic kidney disease (Ovunc et al., 2011, Turner et al., 2003); and TRPV5, involved in calcium transport in the distal convoluted tubule. Combining our current technology with CRISPR/Cas9 gene editing of hPSCs (Freedman et al., 2015) with mutation generation, or correction of (patient-derived) mutations and evaluation of implanted kidney tissues *in vivo* will provide much improved insight into disease mechanisms and allow more focused drug discovery.

Numerous limitations will need to be overcome before a whole functional renal tract can be generated from hPSCs. First, the mammalian kidney receives 20% of the cardiac output, to generate the required high blood flow and hydrostatic pressures within glomerular capillaries, which facilitate glomerular ultrafiltration. Although PSC-kidney precursor cells-derived tissues lack large arteries, in future, placing transplanted cells near arterio-venous loops may enhance the blood supply, as used in transplantation of, e.g., embryonic liver (Fiegel et al., 2010). Second, although the kidney-like tissues that formed contained numerous nephrons, branched collecting ducts were sparse. In future, modulation of *in vitro* protocols to enhance the UB/collecting duct lineage (Taguchi et al., 2014) could be employed. Our study has also gone some way to demonstrating the safety of implanting kidney progenitors *in vivo*: none of the implants showed typical teratoma three-germ-layer differentiation. This is promising for the prospect of using these cells to generate human kidney repair in the future, although many challenges remain including development and survival in a hostile, damaged kidney environment. Evaluation up to a year after transplantation, will be needed to determine the longer-term fates of the cells. Finally, a functional kidney needs to deliver urine into a lower urinary tract. No structures were formed that morphologically resembled either the renal pelvis or the ureter and immunostaining for the urothelial marker uroplakin II was negative (data not shown). In future, therefore, it may be necessary to fuse a host ureter with the kidney tubules that form after implantation of PSC-derived kidney precursors, thus forming a single functional renal tract.

Although there is still room for improvement in the differentiation and maturation efficiency, we have provided proof-of-principle that, when hPSC-derived kidney progenitors are implanted *in vivo*, they can produce more mature kidney structures than in 2D or 3D culture, with near normal patterns of glomerular vascularization. We have generated functional nephrons from hPSCs and shown that their glomeruli are molecularly and ultrastructurally mature. This work greatly advances our progress toward using stem cells for kidney repair and as tools to investigate human genetic diseases affecting the kidney.

## Experimental Procedures

### hPSC Culture and Differentiation

hESC lines, MAN11 and MAN13 (Ye et al., 2017) and HUES1, kind gift from Cowan et al. (2004), were cultured in our feeder-free system modified from that described previously (Baxter et al., 2009). MAN11 and MAN13 were cultured on human Vitronectin (rhVTN-N, Life Technologies, no. A14700) and HUES1 on Matrigel (BD Biosciences, no. 734–1440) substrates, in mTeSR1 or TeSR2 medium (STEMCELL Technologies, nos. 8850 and 5860). Cell differentiation to kidney in 2D cultures was performed as described in Takasato et al. (2014), with a starting cell density of 18,000 cm^−2^, while differentiation in 3D cultures was modified from Takasato et al. (2015). For a detailed description of stem cell culture and differentiation protocols, see Supplemental Information.

### Lentiviral Vector Production and Transduction of hESCs

Construction of the lentiviral shuttle plasmid pRRL.sin.cppt.EF1α-iRFP-E2A-Luc, expressing iRFP and luciferase under the control of the EF1α promoter is described in Supplemental Information. Third-generation lentiviral vectors were constructed as described previously (Dajas-Bailador et al. (2014) and Supplemental Information). HESCs were transduced at an MOI of 5 IU/cell.

### RNA Extraction and Real-Time PCR

RNA samples from cultures were collected at 0, 1, 3, 5, 7, 10, 13, 16, 19, 24, and 30 days after initiation of differentiation. RNA was extracted using the miRVana miRNA isolation kit (Thermo Fisher, AM1560) according to the manufacturer's instructions. Real-time qPCR was performed using the TaqMan RNA-to-Ct 1-Step Kit (Thermo Fisher, 4392653) according to the manufacturer's instructions, on a Bio-Rad C1000 Thermal Cycler fit with a CFX384 Real Time System, using 15 ng of RNA per reaction. The primers used are shown in Table S2.

### Immunostaining of Cultures and Viability/Toxicity Assays

See Supplemental Information and Table S3.

### Implantation of hPSCs and Kidney Progenitor Cells into Immunocompromised Mice

All surgery was carried out under UK Home Office Licence (70/7838) obtained after local ethics committee approval. SCID/beige mice were injected subcutaneously at four sites each, with MAN13 hESCs (1.0 × 10^6^ cells/site) or MAN13-derived kidney progenitors (day 12 or 19 of the differentiation protocol; 3.0 × 10^6^ cells/site). The cells had been previously resuspended in a 2:1 mix of DMEM-F12:Matrigel (see Supplemental Information).

### *In Vivo* Bioluminescence Imaging of Mice, Histology, and Electron Microscopy

See Supplemental Information.

## Author Contributions

I.B., A.S.W., and S.J.K. designed the study. I.B., P.R., F.T., E.S., D.F., P.L., A.P., and Q.W. conducted the research. I.B., P.R., D.F., M.-C.A., R.L., A.S.W., and S.J.K. analyzed the data. I.B., P.R., A.S.W., and S.J.K. wrote the paper. All authors approved the final paper.

## Supplemental Information

Document S1. Supplemental Experimental Procedures, Figures S1–S6, and Tables S1–S3

Movie S1. 3D Reconstruction of a Forming Glomerulus in an Implant from Day 12 PSC-Kidney Progenitors, Related to Figure 5 Note the capillary and mesangial-like cell in the core of the glomerulus.

Document S2. Article plus Supplemental Information
