# Supplementary material for: Generation of Functioning Nephrons by Implanting Human Pluripotent Stem Cell-Derived Kidney Progenitors
Source: Stem Cell Reports. 2018 Feb 8;10(3):766–79. doi: 10.1016/j.stemcr.2018.01.008 (PMC5918196; doi:10.1016/j.stemcr.2018.01.008)
Supplement: Document S1. Supplemental Experimental Procedures, Figures S1–S6, and Tables S1–S3 [file mmc1.pdf]

Stem Cell Reports, Volume *10*

Supplemental Information

Generation of Functioning Nephrons by Implanting Human Pluripotent Stem Cell-Derived Kidney Progenitors

Ioannis Bantounas, Parisa Ranjzad, Faris Tengku, Edina Silajdžić, Duncan Forster, Marie-Claude Asselin, Philip Lewis, Rachel Lennon, Antonius Plagge, Qi Wang, Adrian S. Woolf, and Susan J. Kimber

Figure S1

HUES1

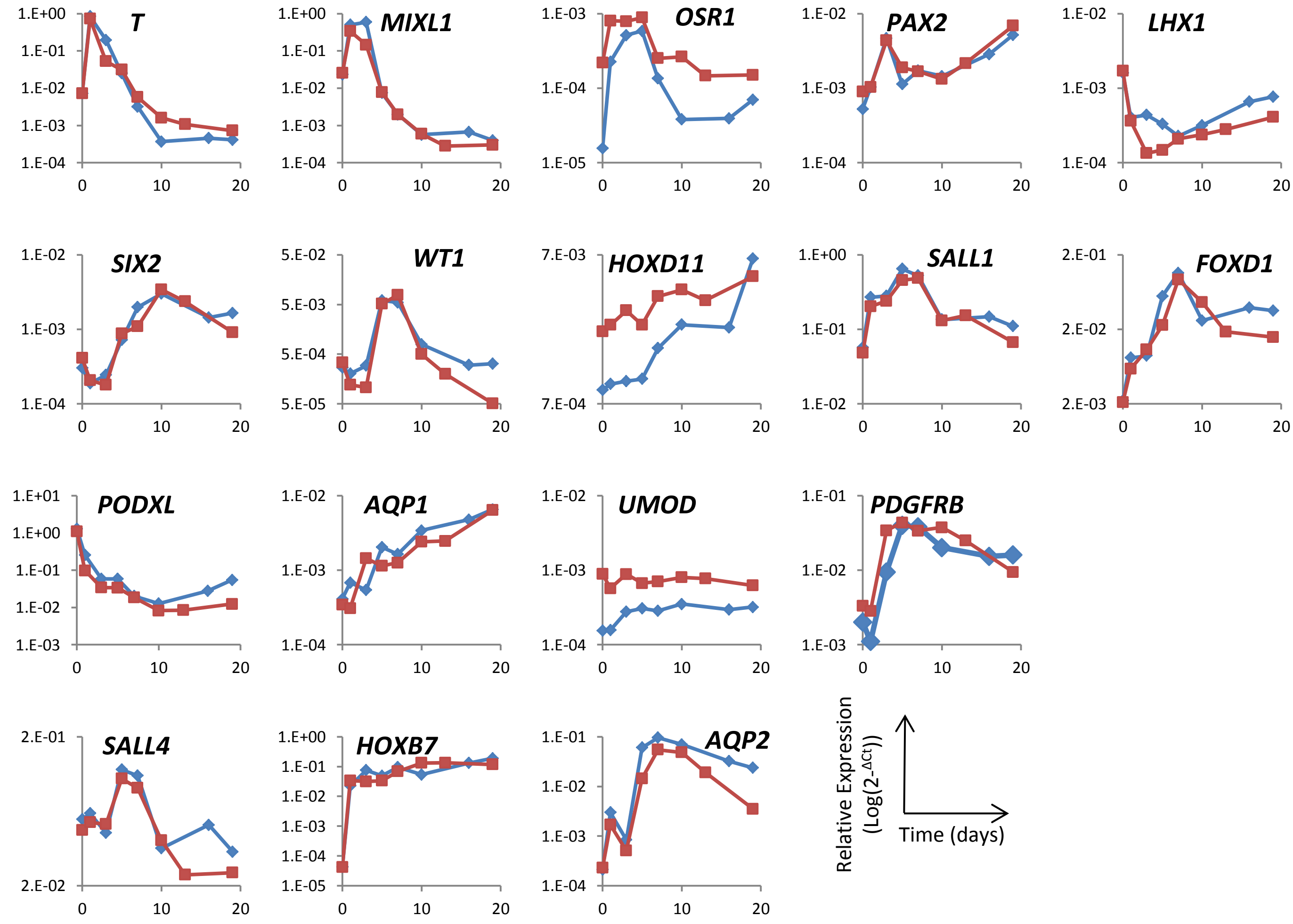

Figure S1 (Continued)

MAN11

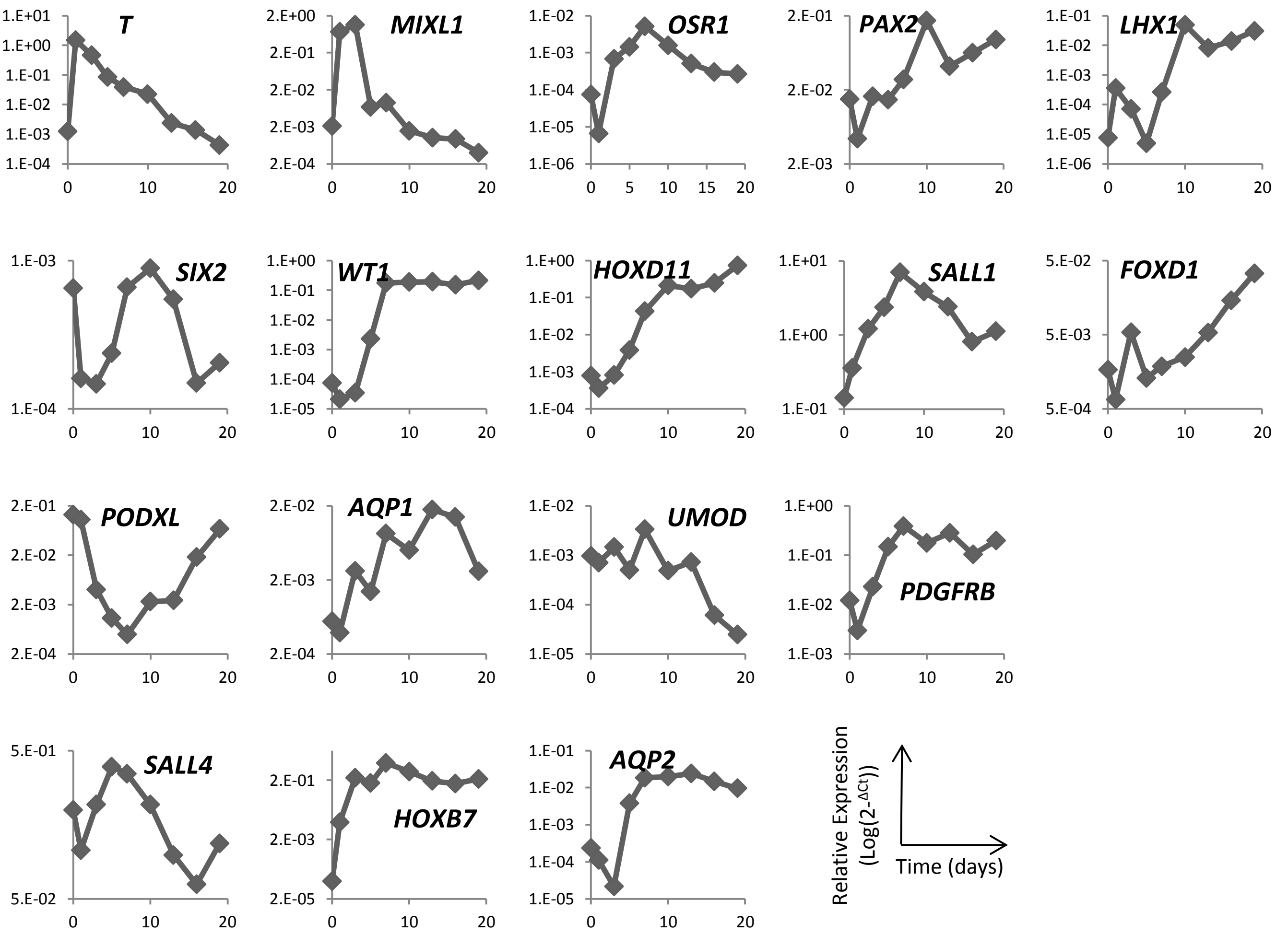

Figure S2

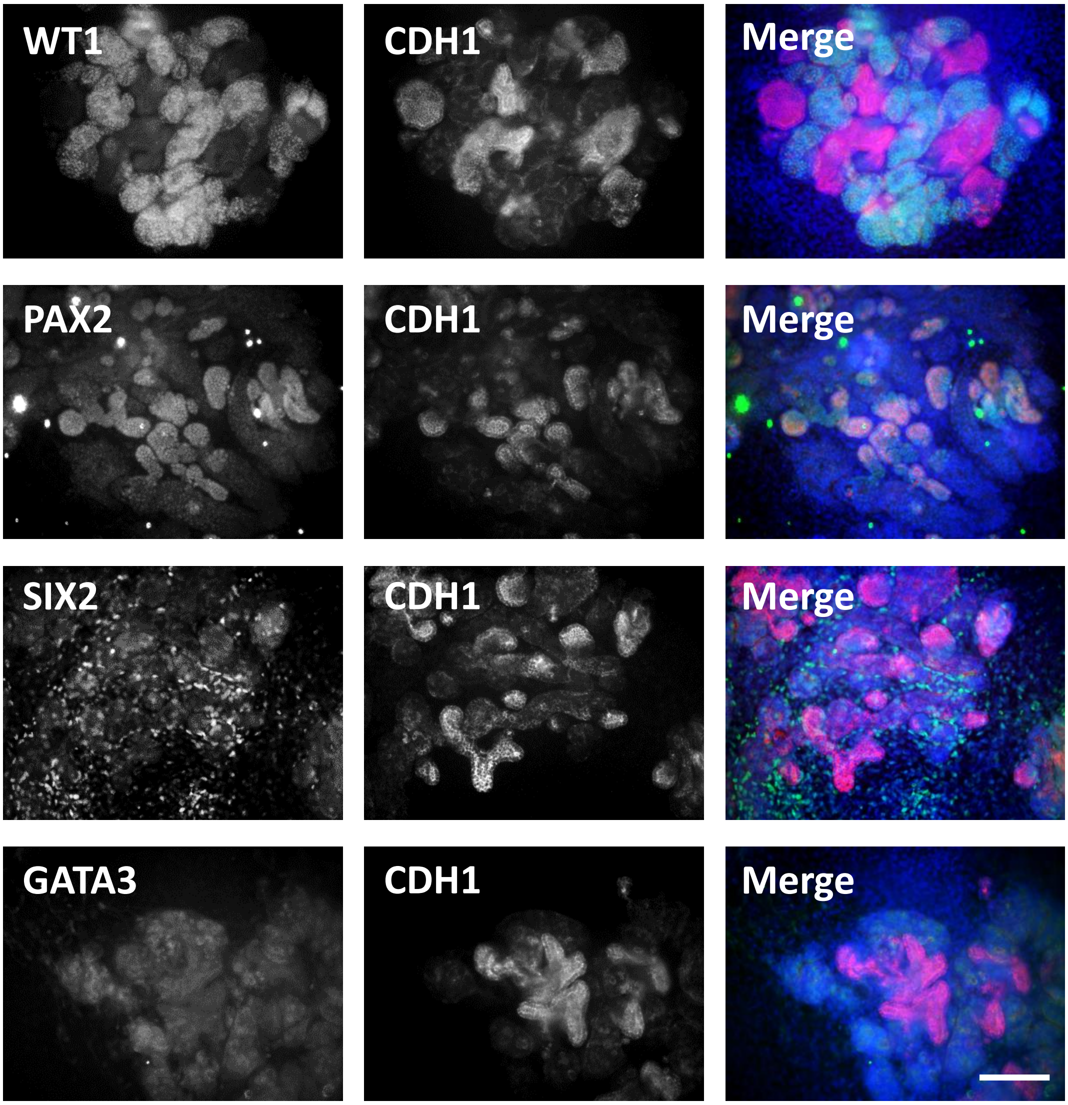

WT1
CDH1
Merge
PAX2
CDH1
Merge
SIX2
CDH1
Merge
GATA3
CDH1
Merge

Figure S3

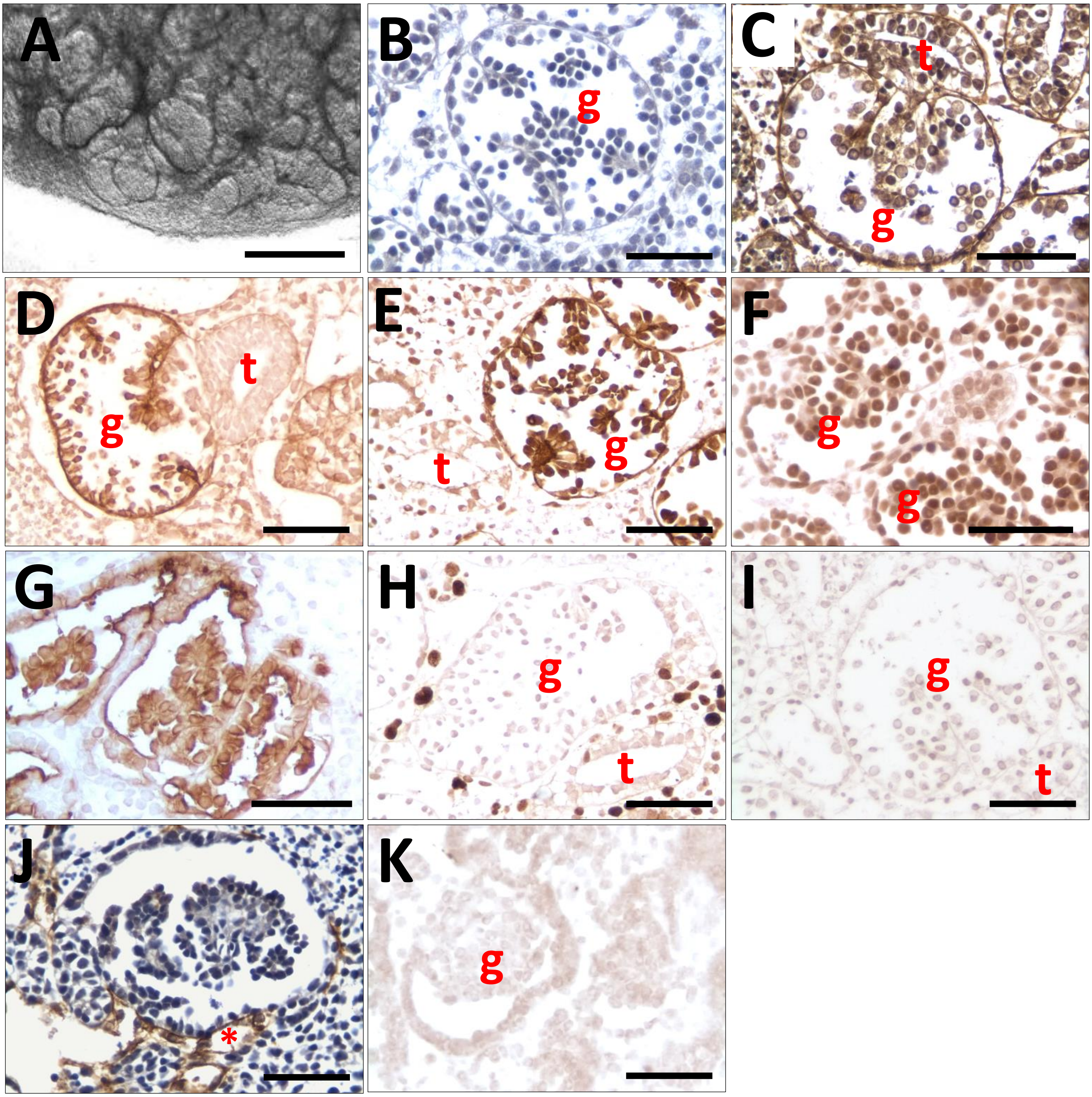

Figure S4

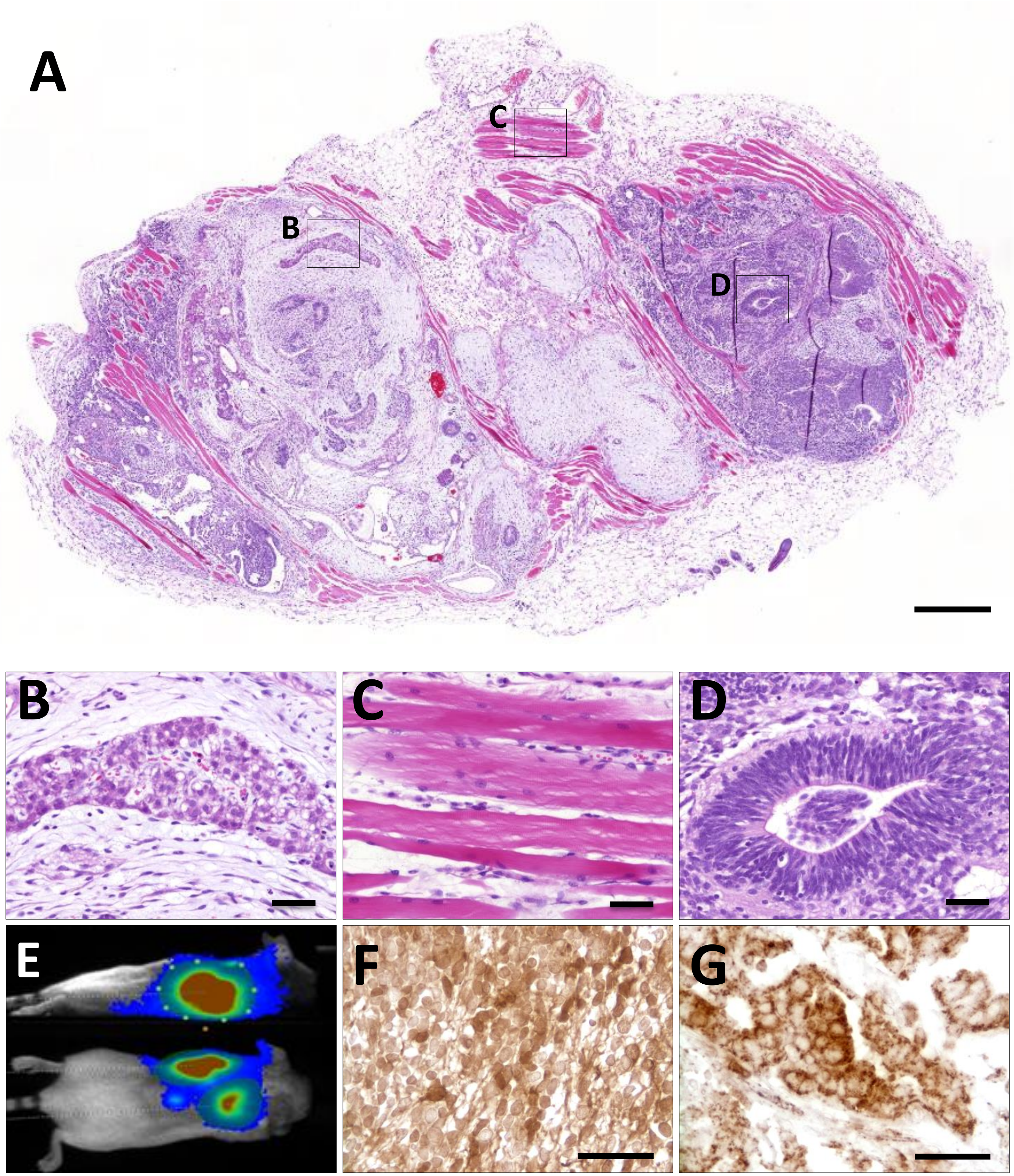

Figure S5

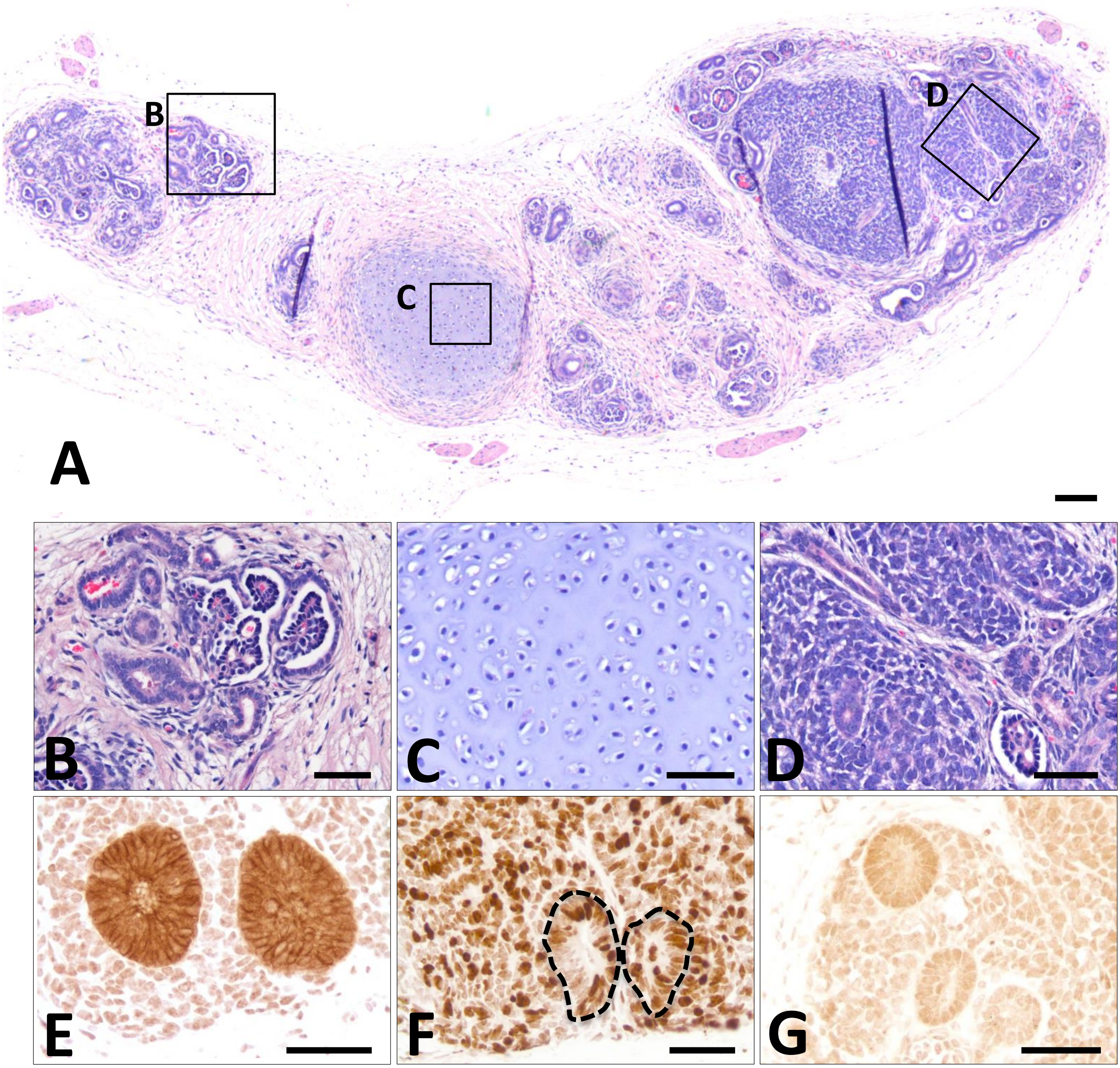

Figure S6

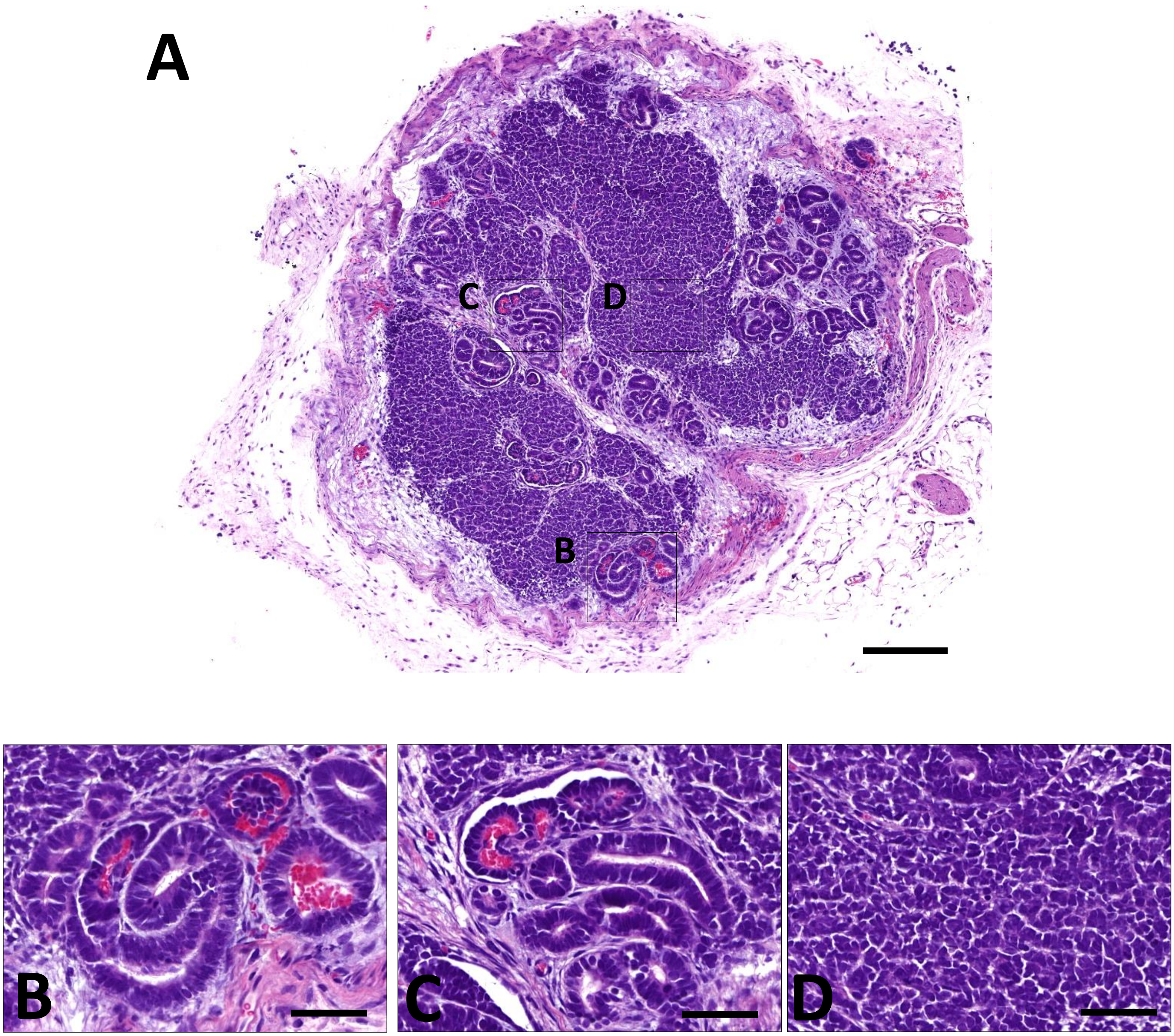

SUPPLEMENTAL FIGURE LEGENDS

Figure S1. (Related to Figure 1). QPCR profiling of HUES1 and MAN11 hPSCs differentiating in the 2D kidney protocol. Cells were differentiated for 19 days and expression of 17 species of transcripts, normalised against GAPDH, was assessed. Biological repeats for HUES1 cells are shown in blue and red.

Figure S2. (Related to Figure 2). Immunocytochemistry of hPSC derivatives after 30 days of 2D culture. Note clusters of nephron progenitors ($WT1^+$) and UB epithelium ($CDH1^+$/$GATA3^+$ and $CDH1^+$/$PAX2^+$), with scattered $SIX2^+$ cells between these clusters. Bar is 40 μm.

Figure S3. (Related to Figure 2). Analyses of hPSC-derived kidney cells in 3D cultures. A. Phase contrast image of the edge of an organoid at the end of the culture period (d25). Note the complex internal structure. **B-K.** Histology sections of organoids, with blue haematoxylin nuclei counterstain in B and J. **B.** IHC for collagen alpha-3 (IV) shows only background (unspecific signal). **C.** IHC for pan-collagen IV shows positive signals (brown) in glomeruli and tubules. **D.** IHC for laminin B2 shows positive signals (brown) in glomerulus but not in an adjacent tubule. **E.** IHC for synaptopodin shows positive cells in glomerular tufts. **F.** Cells in glomerular tufts immunostain for WT1. **G.** Glomerular cells positive for podocalyxin IHC. **H.** IHC for Ki67 detects proliferating cells in the interstitium between a glomerulus and a tubule. **I.** Lack of background signal with anti-rabbit secondary antibody and omission of primary antibody. **J.** PECAM IHC detected capillary-like structures (red asterisk) in the insterstitium near a glomerulus, and around the glomerular capsule, but the glomerular tuft itself is negative. **K.** No significant signal when immunostaining for VEGFA. *g:* glomerular-like structures and *t*: tubules. Bars are 20 μm in A and 50 μm in other frames.

Figure S4. (Related to Figures 3 and 4). Subcutaneous implantation into *beige/SCID* mice of luciferase labelled MAN13 hPSCs. A. Histological overview of teratoma four weeks after implantation stained with haematoxylin and eosin. The teratoma contains a variety of tissues from the three primary embryonic germ layers. The boxed areas indicate: **B.** gland-like endoderm; **C.** muscle mesoderm**;** and **D.** neuro-epithelial-like ectoderm. **E.** Luciferase-labelled implanted cells were detected in living animals four weeks after implantation after luciferin injection and non-invasive imaging by their bioluminescence; dorsal and side views are shown. **F**. Immunostaining of teratomas (brown) for luciferase. **G.** Immunostaining (brown) for human mitochondria. Note that sections in F. and G. are not counterstained. Scale bars: A, 500 μm; B, C, D, F and G 50 μm.

Figure S5. (Related to Figure 4). A kidney-like structure grown from subcutaneously implanted MAN13-derived kidney precursor cells harvested at day 12 in 2D culture. A-D were counterstained with haematoxylin and eosin, while E-G were not counterstained. **A.** Compared with that depicted in Figure 6, this implant appeared less differentiated. **B-D.** Despite clusters of glomeruli and tubules (B), other areas contained cartilage (C) or zones of glomeruli surrounded by mesenchyme-like cells and primitive tubules (D). **E-G.** Within the latter zone, tubules expressed CDH1 (brown in E), both tubules (dotted outlines) and mesenchyme were rich in Ki67 expressing cells (brown in F), and tubules immunostained weakly for PAX2 (brown in G). Scale bars: A, 100 μm; B-G, 50 μm.

Figure S6. (Related to Figure 4). Subcutaneous implantation into *beige/SCID* mice of luciferase-labelled MAN13-derived kidney precursor cells harvested at day 19 in 2D culture. A. Overview. **B and C.** Primitive nephrons. **D.** Undifferentiated mesenchyme-like cells. Compare this tissue mass that contains only very primitive nephrons and tubules with tissues resulting from day 12 implants (see Figure 6). Bars are 100 μm in A and 50 μm in B-D.

SUPPLEMENTAL VIDEO LEGEND

Supplemental Video. (Related to Figure 5). Three D reconstruction of a forming glomerulus in an implant from day 12 PSC-kidney progenitors. Note the capillary and mesangial-like cell in the core of the glomerulus.

Table S1. (Related to Figure 4). Summary of implant experiments.

Cell type injected	# of ani-mals	Sites per animal	# of cells per site	# of sites with cell masses	Diameter of cell mass (cm ± S.E.)	Time of excision (days post-injection ± S.E.)
MAN13 (unlabelled) hESCs	6	4	$1x10^6$	20/24	1.13 ± 0.07	38.8 ± 1.2
MAN13 + LV-iRFP/Luc hESCs	9	4	$1x10^6$	29/36	0.98 ± 0.07	44.6 ± 1.2
MAN13+LV-iRFP/Luc-derived day-12 kidney progenitors (1st run)	8	4	$3x10^6$	9/32	0.61± 0.11	84.3 ± 0.2
MAN13+LV-iRFP/Luc-derived day-12 kidney progenitors (2nd run)	5	4	$3x10^6$	7/20	N/A	79 ± 0.0
MAN13+LV-iRFP/Luc-derived day-19 kidney progenitors	5	4	$3x10^6$	8/20	N/A	84 ± 0.0

Table S2. (Related to experimental Procedures). Sequences of primers used for QPCR experiments.

Primer	Sequence
OSR1 Fwd	CTCCTCGAGATCCGGATTGAG
Rev	GTTCACTGCCTGAAGGAAGG
T Fwd	AGGTACCCAACCCTGAGGA
Rev	GCAGGTGAGTTGTCAGAATAGGT
MIXL1 Fwd	GGTACCCCGACATCCACTT
Rev	GCCTGTTCTGGAACCATACCT
PAX2 Fwd	GCAACCCCGCCTTACTAAT
Rev	AACTAGTGGCGGTCATAGGC
LHX1 Fwd	ATGCAACCTGACCGAGAAGT
Rev	CAGGTCGCTAGGGGAGATG
SIX2 Fwd	CGCCCATGTGGGTCAGTGGG
Rev	AGCCGGGAGCGCTGTAGTCA
HOXD11 Fwd	GCCAGTGTGCTGTCGTTCCC
Rev	CTTCCTACAGACCCCGCCGT
HOXB7 Fwd	GCCTACAAATCATCCGGCCA
Rev	GGTTGGAAGCAAACGCACAA
FOXD1 Fwd	GACTCTGCACCAAGGGACTG
Rev	CCTCGAGCGCGCTAACATAG
GAPDH Fwd	AGCCACATCGCTCAGACAC
Rev	GCCCAATACGACCAAATCC
WT1 Fwd	GGCAGCACAGTGTGTGAACT
Rev	CCAGGCACACCTGGTAGTTT
SALL1 Fwd	AGCGAAGCCTCAACATTTCCAATCC
Rev	AATTCAAAGAACTCGGCACAGCACC
SALL4 Fwd	CAGATCCACGAGCGGACTCA
Rev	CCCCGTGTGTCATGTAGTGA
AQP1 Fwd	ATTAACCCTGCTCGGTCCTT
Rev	ACCCTGGAGTTGATGTCGTC
PODXL Fwd	TCATCATCACCATCGTCTGC
Rev	CCACCTTCTTCTCCTGCATC
AQP2 Fwd	GTGCGCCGAAAATTTCCA
Rev	CCTCGACTTCTCCTTGAAGCA
UMOD Fwd	AACATCACTGATATCTCCCTCCT
Rev	TTGTCTCTGTCATTGAAGCCC
PDGFRB Fwd	GCCGTCAAGATGCTTAAATCC
Rev	TATAGATGGGTCCTCCTTTGGT

Table S3. (Related to experimental Procedures). Antibodies used in immunocytochemical (ICC) and immunohistochemical (IHC) analyses

Antibody	Host	Source	Catalogue #	Application	Dilution
AQP1	Rabbit	Abcam	ab15080	IHC	1:500
CD31 (PECAM)	Mouse	Cell Signalling	3528	IHC	1:100
CDH1 (E-Cadherin)	Mouse	Abcam	76055	ICC	1:300
				IHC	1:1000
Collagen IV	Rabbit	Abcam	ab6586	IHC	1:400
Collagen IVα3	Rat	Chondrex	7076	IHC	1:400
Cubulin	Goat	Santa Cruz	sc-20607	IHC	1:100
Firefly Luciferase	Rabbit	Abcam	21176	IHC	1:10000
GATA3	Goat	R&D Systems	AF2605	ICC	1:200
				IHC	1:200
Human Mitochondria	Mouse	Millipore	MAB1273	IHC	1:200
Ki-67	Rabbit	AbCam	16667	IHC	1:100
Laminin β2	Mouse	Novus Biologicals	NBP2-42387	IHC	1:5000
Nephrin	Sheep	R&D Systems	AF4269	ICC	1:200
				IHC	1:500
PAX2	Rabbit	Thermo-Fisher	71-6000	ICC	1:200
				IHC	1:400
Podocalyxin	Mouse	R&D Systems	MAB1658	IHC	1:200
Podocin	Rabbit	Sigma	P0372	ICC	1:200
				IHC	1:500
PDGFRB	Goat	R&D Systems	AF385	IHC	1:100
SIX2	Rabbit	Proteintech	11561-1-AP	ICC	1:200
Synaptopodin (H-140)	Rabbit	Santa Cruz	sc-50459	IHC	1:200
TRPV5	Rabbit	Abcam	ab137028	ICC	1:200
				IHC	1:500
Uromodulin	Rabbit	Santa Cruz	sc-20631	IHC	1:100
VEGF-A	Mouse	R&D Systems	MAB293	IHC	1:100
WT1	Rabbit	Santa Cruz	sc-192	ICC	1:100
WT1	Rabbit	Calbiochem	CA1026	IHC	1:1000

SUPPLEMENTAL EXPERIMENTAL PROCEDURES

hPSC culture and differentiation

Stem cells were grown on 24-well or 6-well plates coated with 5 μg ml^{-1} recombinant human Vitronectin (rhVTN-N, Life Technologies, #A14700) in the case of MAN11 and MAN13 or with Matrigel BD Biosciences, #734-1440) in the case of HUES1, in mTeSR1 (StemCell Technologies, #5850) or TeSR2 medium (StemCell Technologies, #5860), with the medium changed every two days. The cells were passaged by treatment of the cultures with 0.5mM EDTA solution, pH8 (Invitrogen, #15575-038; diluted in PBS) and replating the cells in mTeSR1 or TeSR2 medium, containing 5nM ROCK inhibitor, Y-27632 (Tocris, #1254) for 24h.

For 2D differentiation, stem cells were plated on vitronectin-coated plates, at a density of 18,000 cells cm^{-2} in mTeSR1 or TeSR2 medium containing 10μM Y-27632. The following day the medium was replaced with STEMdiff™ APEL™ (StemCell Technologies, #05210), containing 8 μM CHIR-99021 (Tocris, #4423) for three days, followed by APEL™ supplemented with 200 ng ml^{-1} FGF9 (Peprotech, #100-23) and 1 μg ml^{-1} heparin (Sigma, #3149) for a further 10 days. Subsequently, the cells were cultured in basal APEL™ medium which was changed daily. Differentiation in 3D cultures was performed according to Takasato et al (2015). Cells were differentiated for the first 7 days as in the 2D protocol above and then dissociated with TrypLE (Life Technlogies, #12605-028). Aliquots of $2.5x10^5$ cells were centrifuged at 400g for 2 min and the pellets were subsequently cultured on a medium-air interface by transfer onto a MilliCell cell culture insert (0.4μm pore size; Millipore, #PICM03050) inserted into the wells of 6-well plates. Upon transfer, the cells were exposed to APEL™ medium containing 5μM CHIR-99021 for 1h, before reverting back to APEL™ with FGF9/Heparin for a further 5 days. Finally, the 3D cultures were maintained in basal APEL™ medium for another 13 days.

Immunostaining of cultures

Cells were washed twice in PBS and then fixed in 4% paraformaldehyde (PFA) for 20 minutes, followed by another two PBS washes. The fixed cells were blocked and permeabilised for 30 min with 3% bovine serum albumin (BSA)/0.3% Triton-X in PBS before overnight incubation at 4˚C with primary antibodies (Table S3) diluted in 3% BSA/PBS. They were then washed three times with PBS/0.1% Triton-X, followed by Alexa-Fluor™-488- or Alexa-Fluor™-594-labelled, species-specific secondary antibodies (Life Technologies; 1:300 dilution in 3% BSA/PBS). Images were collected on a Zeiss Axioimager.D2 upright microscope using a *63x/Plan-neofluar* objective and captured using a Coolsnap HQ2 camera (Photometrics) through Micromanager software v1.4.23. Images were then processed and analysed using *ImageJ* (http://imagej.net/Fiji/Downloads).

Construction of the lentiviral shuttle plasmid

First, the iRFP720 coding sequence was excised from piRFP720-N1 (a gift from Vladislav Verkhusha, Addgene plasmid # 45461; Shcherbakova and Verkhusha, 2013) by EcoRI/XbaI digestion and was cloned into the pHIV-Luciferase vector (a gift from Bryan Welm, Addgene plasmid # 21375), upstream of its IRES element, to obtain the pHIV-iRFP720-IRES-Luc plasmid. To achieve a more efficient translation of the Luciferase coding sequence, the IRES was then replaced with an E2A peptide motif, using a two-step PCR protocol to create the pHIV-iRFP720-E2A-Luc plasmid.
To construct the 3rd generation lentiviral shuttle plasmid pRRL.sin.cppt.EF1α-iRFP-E2A-Luc, the EF1α promoter was first extracted from the pHIV-iRFP-IRES-Luc by PCR, using primers carrying ClaI/XbaI sites at their ends. The PCR product was digested with these enzymes and ligated into ClaI/XbaI-digested pRRL.sin.cppt.CMV-EGFP-WPRE (a gift from James Uney, University of Bristol) to yield pRRL.sin.cppt.EF1α-EGFP-WPRE. Next, pHIV-EF1α-iRFP-E2A-Luc was digested with EcoRI/ClaI, blunt-ended and the EF1α-iRFP-E2A-Luc-containing fragment was ligated into the BamHI/SalI-digested and blunt-ended pRRL.sin.cppt.EF1α-EGFP-WPRE vector.

Lentiviral vector production

Lentiviral vectors were produced by transfection of HEK-293T cells by calcium phosphate precipitation, in 15cm dishes, at 50% confluency, with 10μg pRRL.sin.cppt.EF1α-iRFP-E2A-Luc, 10μg pMDLg-pRRE, 3.4μg pMD2.G and 2μg pRSV-Rev per dish. Cell medium was collected over two days and centrifuged at 6,000g overnight, the pellet resuspended in PBS and the new suspension centrifuged in a SW40-Ti rotor (Beckman Coulter Ltd, High Wycombe, UK) at 50,000g for 90min. Finally, the resulting pellet was resuspended in PBS, at 1:2,000 of the original medium volume. The viral titre was calculated by FACS (detecting iRFP fluorescence) on HEK-293T cells transduced with serial dilutions of the viral preparation. HESCs were transduced with lentivirus at a multiplicity of infection (MOI) of 5 IU/cell.

Viability/Toxicity assays

Cells were plated on vitronectin-coated 24-well plates at $3.5x10^4$ cells per well and grown for six days in mTeSR1 medium, replacing the medium daily. Viability/toxicity assays were then carried out using the ApoTox-Glo™ kit (Promega) according to the manufacturer's instructions. Prior to the start of the assay, the volume of medium in the well was reduced to 200µl and 40µl of the viability/toxicity reagent were added. The cells were replaced in the incubator for 1h, before absorbance was measured directly from the culture plate wells in a GLOMAX Multi+ reader using the $400_{Ex}/505_{Em}$ (viability) and $485_{Em}/520_{Ex}$ (toxicity) filters.

Implantation of hPSCs and kidney progenitor cells into immunocompromised mice

All surgery was carried out under UK Home Office Licence (70/7838) obtained after local ethics committee approval. MAN13 hESCs or MAN13-derived kidney progenitors (day-12 or day-19 of the differentiation protocol) were collected using TrypLE (Life Technlogies, #12605-028) for one or five minutes, respectively, centrifuged at 200 g and resuspended in warm DMEM-F12 culture medium. Cells were then counted and separated into 400 µl aliquots of cold DMEM-F12 containing $6.0x10^6$ or $1.8x10^7$ cells, respectively. Two hundred µl of Matrigel (Becton Dickinson, 356231) was added to the cell suspension prior to injection into 6-week old, female, immunocompromised SCID-Beige mice. Next, 100µl of the final cell suspension (equivalent to $1.0x10^6$ or $3.0x10^6$ cells, respectively) was injected subcutaneously into each of four dorsal sites per mouse, using a syringe fitted with a 23G needle. The first two injection sites were on the left and right shoulder blades and the second two injection sites, approximately 1.5 cm below each of the first two. Prior to injection, the animals were anaesthetised with isoflurane gas.

In vivo bioluminescence imaging of mice

All mice were administered with 100 µL of 10 mg/kg luciferin in PBS intraperitoneally. and some mice were also injected with 100 µL FITC dextran intravenously. The mice were then anaesthetized using 2% isoflurane in O_2 at 2 L/min. before being transferred to the heated imaging bed in order to maintain core temperature. From 5-15 minutes after injection, the mice were scanned in a Biospace Lab PhotonImager Optima (Biospace Lab, Nesles-la-Vallée, France) with the 4D attachment to allow imaging on four sides of the mouse. Mice were culled approximately 1 hour after injection.

Histology and immunohistochemistry

Tissue was fixed in 4% paraformaldehyde, embedded in paraffin and sectioned at 5 µm. Sections were dewaxed and rehydrated, and alternate slides were stained with haematoxylin and eosin (H&E) to assess overall tissue architecture. Images were acquired on a 3D-Histech Pannoramic-250 microscope slide-scanner using a x20 objective (Zeiss) and selected images were captured using the Case Viewer software (3D-Histech). After rehydration, other slides were boiled in an 800W microwave in 10 mM sodium citrate buffer (pH 6.0). After cooling to room temperature, endogenous peroxidase activity was blocked using 0.3% H_2O_2 in PBS for 10 minutes. Sections were permeabilized using 0.2% Triton X-100 (Sigma-Aldrich) for 10 minutes and blocked using 1% bovine serum albumin (BSA) with 10% serum from the species in which the secondary antibody was raised. Sections were incubated overnight at 4°C with the primary antibody + 1% BSA. Primary antibodies used are listed in Table S3. Biotin-conjugated species specific secondary antibodies with 1% BSA were incubated at room temperature for 2 hours. Following PBS washes, slides were incubated in avidin-biotin enzyme complex (Vector Laboratories VECTASTAIN Elite ABC Reagent, PK-6100) for 1 hour at room temperature. Peroxidase activity was detected with the 3, 3'-diaminobenzidine (DAB) peroxidase substrate solution (Vector Laboratories, SK4100) in some cases with haematoxylin counterstain. Sections were dehydrated and mounted with DPX mounting medium and examined under a Leica DMLB 2 microscope. Negative controls omitted primary antibodies. For immunofluorescent imaging, anti-rabbit Alexa 488 (A11034, Thermo Fisher Scientific) and anti-mouse Alexa 594 (A11032, Thermo Fisher Scientific) secondary antibodies and DAPI nuclear stain were used. Sections were mounted with Vectashield antifade mounting medium (H-1000, Vector Laboratories).

Images were collected on a Zeiss Axioimager.D2 upright microscope and captured using a Coolsnap HQ2 camera (Photometrics) through Micromanager software v1.4.23. Specific band pass filter sets for *DAPI, FITC and Texas red were* used to prevent bleed through from one channel to the next. Images were then processed and analysed using *Fiji ImageJ (http://imagej.net/Fiji/Downloads).*For visualisation of the FITC dextran by fluorescent microscopy, dewaxed and rehydrated paraffin embedded sections were mounted with Vectashield antifade mounting medium. Images were acquired on an Olympus IX83 inverted microscope. The images were collected using a *R6* CCD camera with a Z optical spacing of 0.2µm. Raw images were then deconvolved using the Huygens Pro software (SVI).

Transmission electron microscopy and serial block face-scanning *electron microscopy*

Organoid implants were cut into 1 mm cubes and fixed in situ by using 2% (wt/vol) glutaraldehyde (Agar Scientific, UK) in 0.1 M cacodylate buffer (pH 7.2); stained in 1% (wt/vol) osmium tetroxide, 1.5% (wt/vol) potassium ferrocyanide in 0.1 M cacodylate buffer, followed by 1 % (wt/vol) thiocarbohydrazide. After they were stained further in 1% (wt/vol) osmium tetroxide, and soaked in 1% (wt/vol) uranyl acetate overnight. In the final staining step incubation was performed at 60°C with lead aspartate pH 5.5 for one hour (Starborg et al., 2013). Samples were dehydrated in ethanol, infiltrated in TAAB 812 hard resin and sectioned at 70-80nm thickness. Sections were examined using a FEI Tecnai 12G2 Biotwin transmission electron microscope at magnification from 145X to 6800X. For Serial Block face scanning electon microscopy following preparation as above the block was placed in the Quanta 250 FEG (FEI Company) + Gatan 3view system and a 41 μm × 41 μm field of view was chosen and imaged by using a 4096 × 4096 scan, which gave an approximate pixel size of 10 nm. The section thickness was set to 50 nm in the Z (cutting) direction.

SUPPLEMENTAL REFERENCES

Shcherbakova, D.M., and Verkhusha, V.V. (2013). Near-infrared fluorescent proteins for multicolor *in vivo* imaging *Nat Methods 10*, 751-4.

Starborg, T., Kalson, N.S., Lu, Y., Mironov, A., Cootes, T.F., Holmes, D.F., and Kadler, KE. (2013). Using transmission electron microscopy and 3View to determine collagen fibril size and three-dimensional organization. Nat Protoc 8, 1433-1448.
